# Genome-Wide Analysis of *ZmDREB* Genes and Their Association with Natural Variation in Drought Tolerance at Seedling Stage of *Zea mays* L

**DOI:** 10.1371/journal.pgen.1003790

**Published:** 2013-09-26

**Authors:** Shengxue Liu, Xianglan Wang, Hongwei Wang, Haibo Xin, Xiaohong Yang, Jianbing Yan, Jiansheng Li, Lam-Son Phan Tran, Kazuo Shinozaki, Kazuko Yamaguchi-Shinozaki, Feng Qin

**Affiliations:** 1 Key Laboratory of Plant Molecular Physiology, Institute of Botany, Chinese Academy of Sciences, Beijing, China; 2 Graduate University of the Chinese Academy of Sciences, Beijing, China; 3 National Maize Improvement Center of China, China Agricultural University, Beijing, China; 4 National Key Laboratory of Crop Genetic Improvement, Huazhong Agricultural University, Wuhan, China; 5 Signaling Pathway Research Unit, RIKEN Center for Sustainable Resource Science, Suehiro-cho, Tsurumi-ku, Yokohama, Japan; 6 Gene Discovery Research Group, RIKEN Center for Sustainable Resource Science, Suehiro-cho, Tsurumi-ku, Yokohama, Japan; 7 Laboratory of Plant Molecular Physiology, Graduate School of Agricultural and Life Sciences, The University of Tokyo, Yayoi, Bunkyo-ku, Tokyo, Japan; University of Minnesota, United States of America

## Abstract

The worldwide production of maize (*Zea mays* L.) is frequently impacted by water scarcity and as a result, increased drought tolerance is a priority target in maize breeding programs. While DREB transcription factors have been demonstrated to play a central role in desiccation tolerance, whether or not natural sequence variations in these genes are associated with the phenotypic variability of this trait is largely unknown. In the present study, eighteen *ZmDREB* genes present in the maize B73 genome were cloned and systematically analyzed to determine their phylogenetic relationship, synteny with rice, maize and sorghum genomes; pattern of drought-responsive gene expression, and protein transactivation activity. Importantly, the association between the nucleic acid variation of each *ZmDREB* gene with drought tolerance was evaluated using a diverse population of maize consisting of 368 varieties from tropical and temperate regions. A significant association between the genetic variation of *ZmDREB2.7* and drought tolerance at seedling stage was identified. Further analysis found that the DNA polymorphisms in the promoter region of *ZmDREB2.7*, but not the protein coding region itself, was associated with different levels of drought tolerance among maize varieties, likely due to distinct patterns of gene expression in response to drought stress. *In vitro*, protein-DNA binding assay demonstrated that ZmDREB2.7 protein could specifically interact with the target DNA sequences. The transgenic *Arabidopsis* overexpressing *ZmDREB2.7* displayed enhanced tolerance to drought stress. Moreover, a favorable allele of *ZmDREB2.7*, identified in the drought-tolerant maize varieties, was effective in imparting plant tolerance to drought stress. Based upon these findings, we conclude that natural variation in the promoter of *ZmDREB2.7* contributes to maize drought tolerance, and that the gene and its favorable allele may be an important genetic resource for the genetic improvement of drought tolerance in maize.

## Author Summary

Water scarcity is one of the most severe threats to maize production worldwide. Although research has demonstrated that DREB-type transcription factors play important roles in plant water stress response, whether the specific genetic variants in *DREB* genes contribute to plant drought tolerance is largely unknown. Taking advantages of recent technical and methodological advance, we systematically analyzed all the functional *DREB* genes in maize and examined their associations with the natural variation in drought tolerance of 368 maize varieties collected from tropical and temperate regions. A significant association in the *ZmDREB2.7* gene with drought tolerance was detected in that the DNA polymorphisms in the gene promoter region, but not those in the protein coding region, contributed to observed variations in maize drought tolerance, probably due to the distinct gene expression patterns in response to the stress. Overexpressing *ZmDREB2.7* in *Arabidopsis* resulted in enhanced tolerance to drought stress. Moreover, a favorable *ZmDREB2.7* allele, identified from drought-tolerant varieties, was effective in improving plant tolerance to drought stress when it was introduced into a drought-sensitive background. *ZmDREB2.7* and its favorable allele represent a valuable genetic resource for enhancing maize drought tolerance by marker assisted breeding and transformation technology.

## Introduction

Maize is one of the most planted crops world-wide and has tremendous value for providing food, forage, pharmaceuticals, and other industrial products. Its productivity is frequently hampered by water scarcity and so improved drought tolerance is an important goal in many breeding programs. Considerable research has been conducted to better understand the genetic and molecular basis for drought tolerance in plants with the idea that this research will provide information that will greatly increase the efficiency of traditional breeding programs to select for drought tolerance through the use of molecular markers. Alternatively, this research can be used to identify specific genes that can be used to improve drought tolerance using transformation technologies.

Abiotic stress research in *Arabidopsis* has revealed two major ABA-dependent and ABA-independent signaling pathways, that control stress-inducible gene expression. DREBs/CBFs (Dehydration Responsive Element Binding proteins/C-repeat Binding Factors, hereafter referred as DREBs) are thought to be the major transcription factors (TFs) that control stress-inducible gene expression in the ABA-independent pathway [1]. DREB TFs, belonging to the APETALA2/Ethylene-Responsive Factor (AP2/ERF) superfamily of TFs, are able to bind a Dehydration Responsive Element (DRE, core motif: A/GCCGAC, also known as a C-repeat and low-temperature-responsive element [2]–[4], in the promoter region of many drought and/or cold stress-inducible genes. They were first identified using a yeast one-hybrid system to screening for the *trans*-factors of the DRE element identified in a set of drought and cold-inducible gene promoters [5], [6]. There are two groups of *DREB* genes in the *Arabidopsis* genome (*DREB1s* and *DREB2s*) that are composed of six and eight members, respectively [7]. Ectopic or selective expression of *DREB1A/CBF3* can significantly enhance plant tolerance to multiple abiotic stresses, including drought, freezing and high salinity [6], [8]. Over-production of a constitutive active form of DREB2A (DREB2A-CA) protein conferred significant both drought and heat tolerance in transgenic plants [9], [10]. Thus, distinct from DREB1, post-translational modification of the DREB2A protein was demonstrated to finely modulate its abundance and activity [11].

In plants, the *DREB* gene family consists of multiple genes. Although they are primarily involved in the regulation of water-stress-related gene expression, other functions have been noted for specific *DREB* genes. For example, *DREB1D/CBF4* plays a role in plant drought stress tolerance which is in contrast to the homologous *DREB1A/CBF3* gene that functions in cold response [12]. *DREB1C/CBF2* has been characterized as a negative, but not a positive, regulator of plant cold stress response by tightly controlling *DREB1A/CBF3* and *DREB1B/CBF1* expression [13]. *DREB2C* has been reported to play a role in heat rather than drought tolerance [14]. The functional divergence of different *DREB* genes has proven to be an attractive and challenging topic of research.

Studies in other species, such as rice, tomato, soybean, wheat, barley and maize, suggest that *DREB* genes play a central role in plant stress response [15], [16]. In maize, two *DREB* genes (*ZmDREB1A* and *ZmDREB2A*) belonging to the DREB1 and DREB2 subgroups, respectively, were cloned and demonstrated to be upregulated in response to plant water stress [17], [18]. It was found that, distinct from *Arabidopsis DREB2A*, *ZmDREB2A* gene expression in response to abiotic stress was regulated *via* an alternative splicing mechanism and that the expressed protein could directly activate downstream gene expression [18]. Similar findings in rice, wheat and barley, indicate the presence of a mechanism that finely modulates the activity of stress-inducible TF genes and suggest that the molecular mechanism is different in monocot and dicot plants [19]–[21]. Other homologous *DREB* genes in maize have not been identified and characterized.

Although *DREB* genes play an important role in plant response to water stress, several important questions remain and require further research. For instance: is the natural variation in *DREB* genes directly associated with levels of drought tolerance in a plant; which *DREB* gene is the most important for the genetic improvement of drought tolerance; can a favorable allele or alleles of a key *DREB* gene be identified in order to facilitate molecular breeding programs which aim to select for drought tolerance? Answering these questions will not only facilitate the genetic improvement of drought tolerance but will also increase our knowledge of the biological function of this gene family. With the completion of the sequencing of the maize B73 genome, it is now possible to identify all maize *DREB* genes and systematically evaluate their contribution to drought tolerance. Association studies, based on genetic disequilibrium linkage (LD), provide a novel approach for dissecting complex trait loci in plants [22]–[25]. Moreover, maize is thought to be an ideal plant species due to its high level of genetic diversity and quick LD decay, which was estimated to be within several kilobases (kbs) among maize landraces [26]. This feature makes the resolution of genome-wide association studies (GWAS) more precise at the gene level than that in self-pollinated plant species, provided that high-density and genome-wide DNA markers are available [27], [28]. Candidate gene association analysis is made possible with high-throughput technology which enables the discovery and detection of DNA polymorphisms (e.g. Single Nucleotide Polymorphism, SNP) and ensures that markers are within or closely-linked to genes contributing to complex traits [27]. Therefore, this strategy has been widely used in human and animal systems and successfully applied to detect allelic diversity of genes controlling alpha-tocopherol and β-carotene content, aluminum tolerance, kernel size, and fatty acid content in maize and/or rice, given that proper statistic models were employed [29]–[36]. Additionally, after genome-scale resequencing large numbers of varieties with different genetic backgrounds, GWAS accelerates the genetic dissection of complex traits in crops [37]–[39].

In the present research, eighteen maize *ZmDREB1* and *ZmDREB2* genes were cloned and analyzed to determine their phylogenetic relationship, chromosomal synteny with rice and sorghum, pattern of gene expression in response to drought stress, and their protein transactivation activity. Importantly, the association between the genetic variation of each *ZmDREB* gene with drought tolerance was evaluated using a diverse population of maize consisting of 368 varieties from tropical and temperate regions. A strong association between *ZmDREB2.7* gene sequence variance and the degree of drought tolerance at seedling stage was detected. Differences in the promoter region of *ZmDREB2.7*, but not the protein coding region itself, was associated with distinct patterns of gene induction in response to drought stress in the different maize varieties. Moreover, a favorable allele of the *ZmDREB2.7* gene was identified in drought-tolerant varieties.

## Results

### Cloning and Phylogenetic Analysis of *ZmDREB* Genes

In order to identify all genes encoding *ZmDREBs* in maize, multiple searches were first performed for all maize genes encoding AP2/ERF TFs using various plant TF databases. The corresponding sequences were then downloaded from the 5b.60 version of the maize genome sequence database (http://www.maizegdb.org/). Additionally, *DREB* orthologous genes from *Arabidopsis*, rice and sorghum were also identified and downloaded. All of the resultant sequences were then pooled and redundancies were eliminated. Every sequence was manually examined to determine the number and exact location of the AP2/ERF DNA domains. In total, 210 proteins containing AP2/ERF domain(s) were identified in the maize B73 genome (Table S1). Based upon the phylogenetic classification of this superfamily in *Arabidopsis* [7], 44 of the maize proteins, containing multiple AP2/ERF domains or lacking a conserved WLG motif within the domain, were classified in the APETALA2 subfamily, which is most likely involved in floral organ development. Three of the proteins, containing both AP2 and B3 domains, were classified in the RAV subfamily. Of the remaining 163 proteins, possessing only one AP2 domain, 65 members were classified in the DREB (A) subfamily and 98 members were placed in the ERF (B) subfamily (Table S2).

The canonical DREB proteins belong to the A-1 (DREB1) and A-2 (DREB2) subgroups within the DREB subfamily. Ten maize genes belonging to each of these subgroups were identified. Due to our interest in these *DREB* genes, we attempted to clone them from the B73 inbred line of maize. As a result, 18 genes were successfully cloned, and a sequence analysis of the cloned genes showed that they were 100% identical to the annotated gene sequences. All the genes are intronless except that *ZmDREB2.2* and *ZmDREB2.1/2A* [18] contain one and two introns, respectively. Two of the 20 identified genes, *GRMZM2G323172* and *GRMZM2G348307*, containing multiple introns, failed to be obtained from the maize cDNA libraries prepared from various normal growing or stressed tissues collected at different developmental stages.

**Figure 1:**
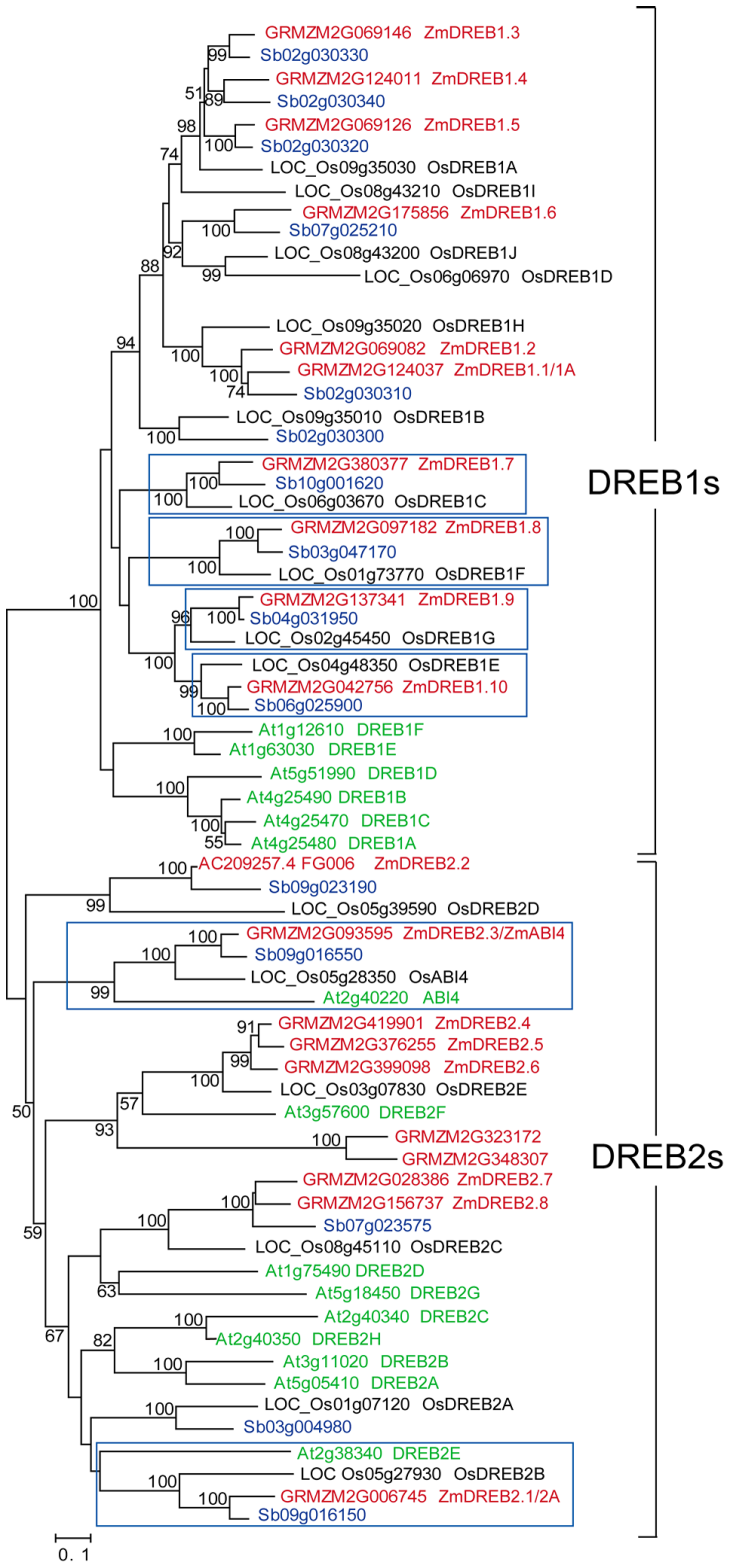
Phylogenetic tree of canonical *DREB1* and *DREB2* genes in maize, rice, sorghum and *Arabidopsis*. The phylogenetic tree was constructed based on the sequence alignments of sixty-six, full-length *DREB* genes from four species. The gene codes and names are illustrated in red for maize; black for rice; blue for sorghum; and green for *Arabidopsis*. The gene names used for *AtDREBs*, *OsDREB1s* and *OsDREB2s* were according to Sakuma et al., 2002 [7], Mao et al., 2012 [65], and Matsukura et al., 2010 [19]. Genes contained within a blue-box were considered to be direct orthologous genes across species. Bootstrap values from 1,000 replicates were indicated at each node and the scale represents branch lengths.

A phylogenetic tree of the ZmDREB proteins and their orthologs from rice, sorghum and *Arabidopsis* was constructed (Figure 1; Table S3). The previously cloned genes encoding ZmDREB1A and 2A proteins [17], [18] were renamed as *ZmDREB1.1* and *2.1*, respectively. The DREB1 group consists of 36 proteins, ten each from maize, sorghum and rice, and six from *Arabidopsis*. Interestingly, the proteins derived from monocots clustered separately from those of dicots. Some proteins from three of the monocot plants examined displayed pairwise correspondences with high bootstrap support, suggesting that these genes are phylogenetically conserved across these species. Furthermore, proteins from maize and sorghum shared a closer phylogenetic relationship than those from maize and rice, which is consistent with the concept that sorghum is a closer relative of maize than rice. The DREB2 group consists of 30 genes, 10, 5, 6 and 9 from maize, sorghum, rice and *Arabidopsis*, respectively. The ABI4 orthologs identified in these four species were found in the DREB2 group and formed a clade. Compared to the DREB1 group, proteins in the DREB2 group are more phylogenetically divergent, with the exception of the ABI4-type proteins that are conserved across all four of the examined species. *ZmDREB2.4, 2.5, 2.6* and *ZmDREB2.7* and *2.8* most likely represent duplicated genes in maize since only one ortholog of each of these genes could be found in the rice and sorghum genomes. The two genes that failed to be cloned clustered together and orthologs could not be identified in the other species. Based upon these results, it is possible that they are pseudogenes that may have originated as a result of genome duplication in maize and subsequently became dysfunctional over the course of evolution. In summary, ten *ZmDREB1* and eight *ZmDREB2* genes, including *ZmDREB2.3/ZmABI4*, were cloned from the B73 inbred line of maize.

### Segmental Duplication of *ZmDREB* Genes in Rice, Sorghum and Maize Genome

**Figure 2:**
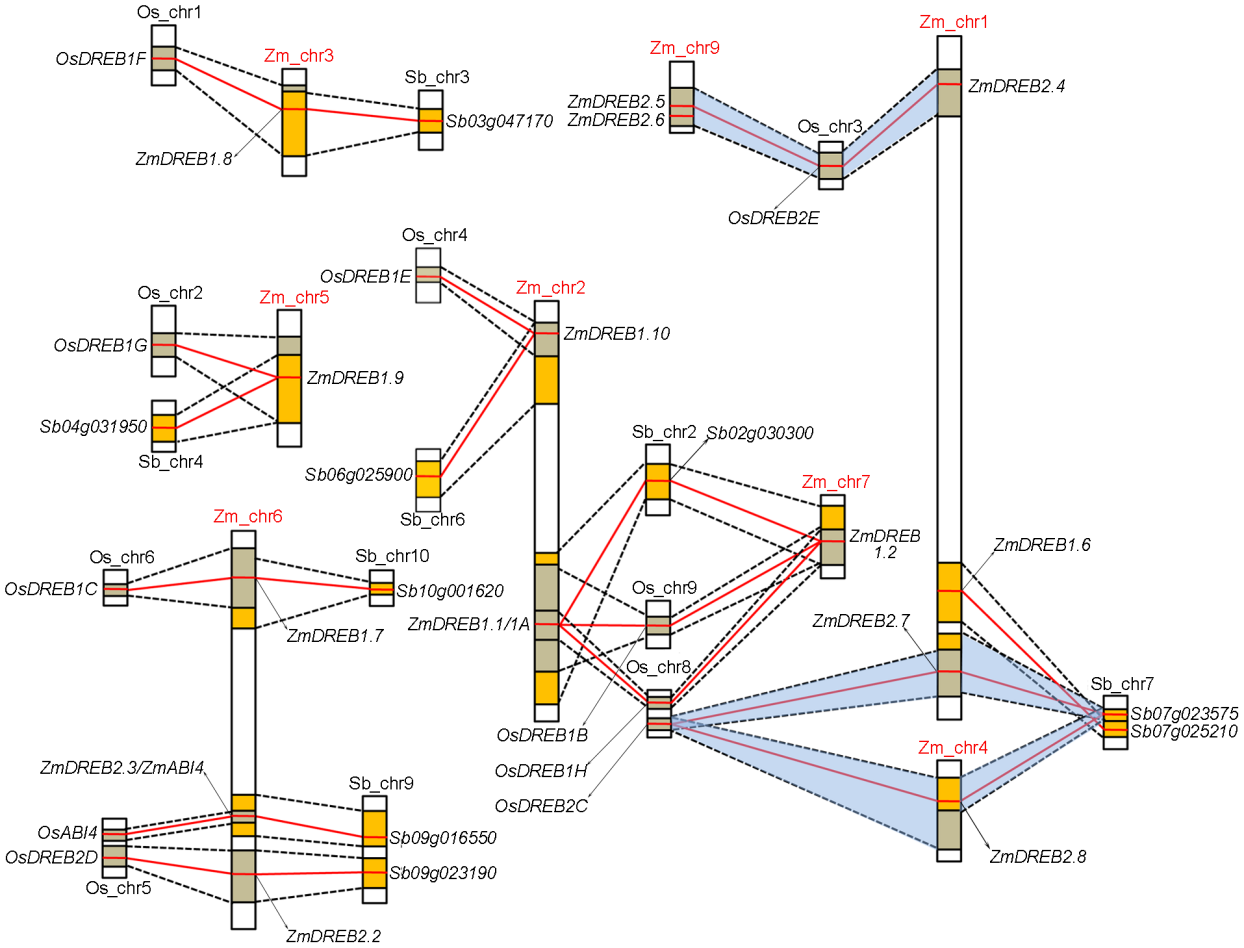
Synteny of chromosomal segments containing *ZmDREB* genes among rice, sorghum and maize genomes. The maize, sorghum, and rice genomes are abbreviated as *Zm, Sb, Os*, respectively. Homologous chromosome segments between the different genomes are linked by black dotted lines. Each *DREB* orthologous gene pair is connected by a red line. Yellow boxes indicate homologous regions between the maize and sorghum genomes while gray boxes identify homologous regions in the maize and rice genomes. The blue shaded regions indicate two segmental duplications in the maize genome, corresponding to one rice and/or sorghum segment.

Since comparative genomic study of gene synteny is indicative of homologous gene function exploration, the colinearity of this gene group within rice, sorghum, and maize genome was explored. Gene colinearity data were collected from the Plant Genome Duplication Database (PGDD, http://chibba.agtec.uga.edu/duplication, Table S4) using *ZmDREB* genes as anchors. Each genomic syntenic block was defined as the chromosomal segment consisting of multiple homologous genes across species [40]. Genes located on chromosomal segments containing *ZmDREB* genes shared good synteny with those in rice and sorghum, especially for *ZmDREB1.7, 1.8, 1.9, 1.10, 2.2* and *ZmDREB2.3/ZmABI4* (Figure 2). This indicates that not only the individual genes but these entire chromosomal segments are evolutionally conserved. Two segments on chromosomes 8 and 9 of rice, containing *OsDREB1B* and *1H*, share synteny with two segments in maize on chromosomes 2 and 7, carrying *ZmDREB1.1/1A* and *1.2*, respectively. Interestingly, their orthologs in sorghum are tandem duplicated on a single chromosomal segment. One rice chromosomal block containing *OsDREB2C* shared synteny with two segments on chromosomes 1 and 4 of maize with its orthologous genes, *ZmDREB2.7* and *2.8*, on each of them. However, only one syntenic block can be found in the sorghum genome (Figure 2). Another fragment on chromosome 3 of rice containing *OsDREB2E* is also duplicated on chromosomes 1 and 9 of maize, probably serving as the origin of *ZmDREB2.4 and 2.5*. Additionally, *ZmDREB2.5* has a tandem duplicated gene, *ZmDREB2.6*. Although the syntenic segment in sorghum can be identified, *SbDREB* orthologs could not be found. Based on the collective data, it appears that most of the *DREB1* genes existed prior to the divergence of the rice, sorghum and maize genomes, due to the genetic synteny across species; however, some *ZmDREB2* genes may have originated from the allotetraploid origin of the maize genome and tandem duplication.

### Expression Profiles of *ZmDREB* Genes

**Figure 3:**
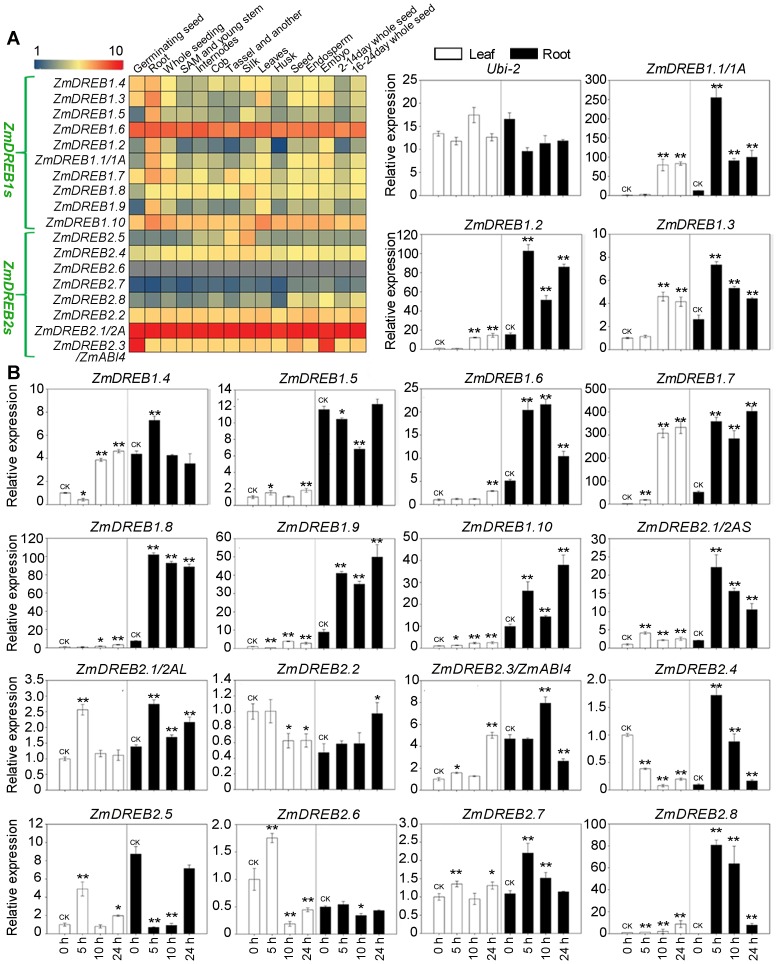
Expression profiles of eighteen *ZmDREB* genes. (A) A heat map illustrating levels of gene expression level of the 18 *ZmDREB* genes in fifteen different tissues from various developmental stages. Normalized gene expression values are shown in different colors that represent the levels of expression indicated by the scale bar. The gray color represents unavailable data. (B) Relative levels of gene expression of 18 *ZmDREBs* in maize B73 leaf and root tissue grown under normal and drought conditions. The *ZmUbi-2* gene, which is constitutively expressed under a wide array of conditions, was used as an internal control. For leaf tissue, the collection time points, 5, 10, and 24 hours, reflected relative leaf water content (RLWC) of 70%, 60% and 58%, respectively. For the sampled root tissues, dehydration stress was applied to hydroponically cultured seedlings for 5, 10, and 24 hours, and RLWC was determined to be approximately 70%, 60% and 58% at the corresponding time points, respectively. Seedlings for the leaf studies were grown and drought stressed in soil (see Materials and Methods). Data represent the mean ± SD of three biological replicates. (T-test, *p<0.05, **p<0.01).

In order to better understand the function of each of the *ZmDREB* genes, their expression profiles were investigated in 15 different tissues of maize plants growing under non-limiting growth conditions. Using transcriptomic data from maize B73 [41], an expression heatmap was constructed for 17 *ZmDREBs* plus *ZmDREB2.3/ZmABI4* in different tissues from 15 developmental stages (Figure 3A). Results indicated that the expression patterns of different *ZmDREB* genes varied greatly. Transcripts of *ZmDREB1.6, 1.10, 2.1, 2.2, and 2.4* were constitutively expressed in the various tissues. *ZmDREB1.6* and *2.1* showed a relatively high level of expression compared to the other *ZmDREB* examined. This result is consistent with a previous report that *ZmDREB2.1/2A* gene activity is regulated by stress-induced alternative splicing and that non-functional transcripts are abundant under non-stress conditions [18]. Evidently, the transcripts of *ZmDREB2.3/ZmABI4* were highly present in germinating seeds and embryos. Additionally, all *ZmDREB1*-type genes were found to be relatively highly expressed in roots, and other *ZmDREB2* genes exhibited a constitutively low level of expression in different tissues in the B73 variety grown under non-limiting conditions.

Next, the expression of all the *ZmDREB* genes was experimentally examined in leaves and roots of 3-week-old drought-stressed maize seedlings by quantitative real-time PCR analyses. As illustrated in Figure 3B, in the genotype of B73, a dramatic upregulation of all *ZmDREB1* genes was observed in response to dehydration, especially in the roots. The greatest dehydration-inducible gene response was observed in *ZmDREB1.7*, whose expression was upregulated more than 400-fold in roots, and 300-fold in leaves, relative to expression under normal growing conditions. The induction response of *ZmDREB2* genes was lower than that of *ZmDREB1* genes. Among the *ZmDREB2* genes, only *ZmDREB2.8* exhibited the highest dehydration-inducible response with about an 80-fold increase in root expression, however, it was not greatly dehydration-inducible in leaves. A clear induction of *ZmDREB2.3/ZmABI4* gene was observed in dehydrated leaves, even though this gene was shown to be mainly expressed in embryos and during germination under non-stressful growth conditions. The expression of *ZmDREB2.7* was only slightly upregulated in response to dehydration, exhibiting about a 1.4-fold and 2.0-fold increase in transcript abundance in drought-stressed leaves and roots, respectively. In contrast to the other *ZmDREB* genes, expression levels of *ZmDREB2.2* and *ZmDREB2.4* decreased in leaves in response to dehydration. Collectively, the data indicate that different *ZmDREB* genes exhibit variable levels of expression in different tissues and developmental stages of maize, as well as in response to dehydrative stress. These data suggests that *ZmDREB* genes play diverse roles in maize development and stress response.

### Transactivation Activity of *ZmDREB* Genes

**Figure 4:**
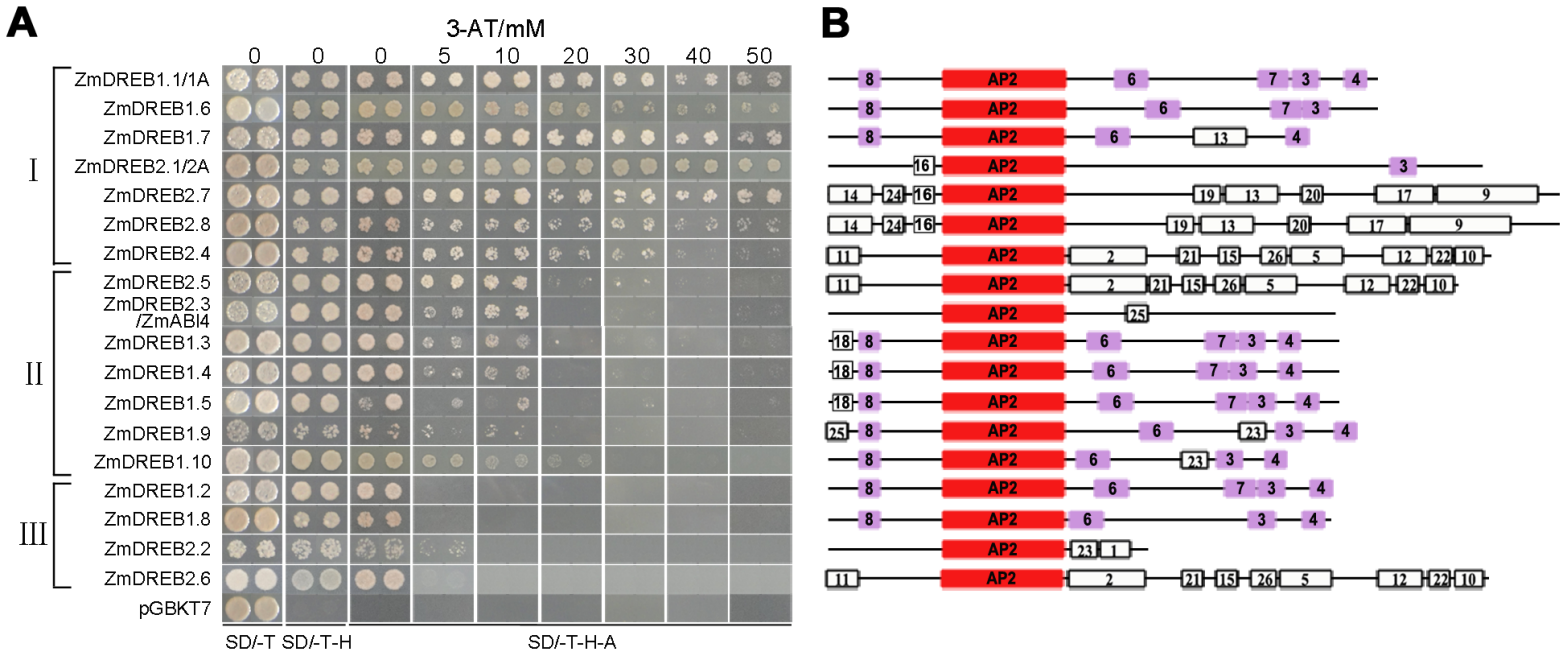
Transactivation activity assay and motif analysis of 18 ZmDREB proteins. (A) Cultures of the AH109 yeast, transformed with a plasmid containing different *ZmDREB* genes, were diluted and inoculated on to a synthetic dropout (SD) media without tryptophan (SD/-T), without tryptophan and histidine (SD/-T-H), or without tryptophan, histidine, and adenine (SD/-T-H-A). The culture plates were amended with different concentrations of 3-aminotriazole (3-AT). Genes were classified into one of three groups (I, II, or III) representing high, medium, or low transactivation activity, respectively. Photos were taken 2 days after inoculation for the plates without 3-AT, and 5 days after inoculation for the plates with various concentrations of 3-AT. (B) Motif analysis of ZmDREB proteins. Conserved protein motifs were identified using the SALAD database (http://salad.dna.affrc.go.jp/salad/). Different motifs were numbered from 1 to 26 and genes labeled with the same number(s) indicate that the same motif(s) was present in the different ZmDREB proteins. The conserved AP2/ERF domain is highlighted in red. Motifs labeled in purple were specific to ZmDREB1 group proteins, except that Motif 3 was also identified in the ZmDREB2.1/2A protein.

DREB proteins function as transactivators that regulate the transcription of downstream target genes in response to abiotic stress. The transactivation activity of each ZmDREB protein was characterized using a yeast activation assay. All eighteen genes were subcloned into a yeast expression vector in a fusion of GAL4-DNA binding domain and transformed into yeast reporter cells which harbor a reporter gene, *HIS3*, driven by the GAL4 upstream activating sequence. The level of transactivation activity was measured by the ability of the transformed yeast cells grow on a stringent selective medium containing 0–50 mM 3-aminotriazole (3-AT), which is a competitive inhibitor of HIS3 protein. Results indicated that the ZmDREB proteins can be classified into three groups based upon their levels of transactivation activity (Figure 4A). Three ZmDREB1 (1.1/1A, 1.7, 1.6) and four ZmDREB2 (2.1/2A, 2.4, 2.7, 2.8) proteins exhibited the highest level of transactivation activity. Five ZmDREB1 (1.3, 1.4, 1.5, 1.9, 1.10), ZmDREB2.5, and ZmDREB2.3/ZmABI4 proteins exhibit moderate levels of transactivation activity as determined by their ability to grow well on the selective medium amended with 10 mM 3-AT. Lastly, four ZmDREB1 (1.2, 1.8, 2.2, and 2.6) proteins exhibited minimal transactivation activity as the yeast cells transformed by these plasmids could only grow on a medium without 3-AT.

In order to gain insight into the differences in transactivation activity exhibited by the ZmDREB proteins, the sequence similarity between all of the proteins was examined (Figure 4B). In addition to the conserved AP2/ERF DNA-binding domain, all of the ZmDREB1s proteins commonly shared a number of conserved motifs, such as motifs 3, 4, 6, 7 and 8. The sequences of ZmDREB2 proteins, however, were more diversified in relative comparison to ZmDREB1 proteins. ZmDREB2.1/2A contained motif 3 which was present only in DREB1 proteins but absent in the other ZmDREB2 proteins. ZmDREB2.7 and 2.8, which displayed high transactivation activity, shared a similar motif structure. Motif 13, found in these two proteins, was also present in ZmDREB1.7 but not in any other proteins. Although the motif composition of ZmDREB2.4, 2.5 and 2.6 were highly conserved, the protein transactivation activity of ZmDREB2.6 was much lower than ZmDREB2.4 and 2.5. Therefore, other unconserved regions or some key amino acid residues of these proteins may be responsible for the observed differences in protein activity. ZmDREB2.3/ZmABI4 and ZmDREB2.2 share little similarity to the other ZmDREB proteins. These results demonstrated that transactivation activity and motif organization among the different ZmDREB proteins were remarkably distinctive. Taken together with the diverse patterns of gene expression exhibited by these genes, it suggested that *ZmDREB* genes in maize may have very diversified functions.

### Association Analysis of Natural Variation in *ZmDREB* Genes with Maize Drought Tolerance

Previous research reported that ZmDREB1.1/1A and 2.1/2A are transcription factors that play an important role in the regulation of maize drought-stress response [17], [18]. In order to further investigate whether the natural variation in any of the genes encoding ZmDREB1 and 2 TFs was associated with the diversity in drought tolerance of maize varieties, an association analysis was conducted for each of the *ZmDREB* genes. Recently, 525,105 high-quality maize SNP markers (minor allele frequency (MAF)≥0.05) were identified from transcriptomic sequencing of a maize natural diversity panel consisting of 368 inbred lines from tropical and temperate regions [39], [42]. These markers were then utilized to characterize the presence of genetic polymorphisms in each of these 18 *ZmDREB* genes. Among the *ZmDREB* genes, 14 were found to be polymorphic with 17 SNPs on average identified in each gene. The polymorphic information was currently absent for four genes, due to above 60% missing rate in the genotyping data. *ZmDREB2.1/2A* was found to be the most polymorphic, with 42 SNPs in this natural diversity panel. The drought stress tolerance of each variety was also investigated. The survival rate of seedlings under severe drought conditions was scored. Statistically, the inbred lines from tropical regions exhibited higher survival rates in comparison to those from temperate regions or B73 derivatives (Figure S1; Table S5). These data supported the hypothesis that varieties existing within the area of origination may possess better and wider resistance than those in cultivated regions. Three kinds of statistical models were applied to identify significant genotypic and phenotypic associations. Specifically, a general linear model (GLM), principle component analysis (PCA), and a mixed linear model (MLM) were used in the associations. PCA was applied to correct for spurious associations caused by population structure. MLM incorporated both PCA and a Kinship matrix (to correct for the effect of cryptic relatedness) and was considered to be effective for controlling false positives in the association analysis [42]–[45]. The analysis detected significant associations in the genetic variation in *ZmDREB2.7* and *ZmDREB2.3/ABI4* under different models. However, *ZmDREB2.7* was the gene that was the most significantly associated (−logP = 3.07) with drought tolerance in this natural variation panel (Table 1, Figure S2).

**Table 1:** Association analysis of natural variation in *ZmDREB* genes with drought tolerance at seedling stage in the maize diversity panel.

Gene ID	Gene Name	Chr.	Polymorphic number*	GLM	PCA	PCA+K	
				p< = 0.01	p< = 0.01	p< = 0.01	p< = 0.001
GRMZM2G042756	*ZmDREB1.10*	2	18	0	0	0	0
GRMZM2G069082	*ZmDREB1.2*	7	10	0	0	0	0
GRMZM2G069146	*ZmDREB1.3*	7	20	0	0	0	0
GRMZM2G124011	*ZmDREB1.4*	2	23	0	0	0	0
GRMZM2G069126	*ZmDREB1.5*	7	25	0	0	0	0
GRMZM2G175856	*ZmDREB1.6*	1	3	0	0	0	0
GRMZM2G380377	*ZmDREB1.7*	6	20	0	0	0	0
GRMZM2G097182	*ZmDREB1.8*	3	-	-	-	-	-
GRMZM2G137341	*ZmDREB1.9*	5	4	0	0	0	0
GRMZM2G124037	*ZmDREB1.1/1A*	2	22	0	0	0	0
AC209257.4_FG006	*ZmDREB2.2*	6	1	0	0	0	0
GRMZM2G093595	*ZmDREB2.3/ZmABI4*	6	33	2	1	1	0
GRMZM2G419901	*ZmDREB2.4*	1	-	-	-	-	-
GRMZM2G376255	*ZmDREB2.5*	9	-	-	-	-	-
GRMZM2G399098	*ZmDREB2.6*	9	-	-	-	-	-
GRMZM2G028386	*ZmDREB2.7*	1	28	6	1	1	1
GRMZM2G156737	*ZmDREB2.8*	4	1	0	0	0	0
GRMZM2G006745	*ZmDREB2.1/2A*	8	42	0	0	0	0

* MAF (Minor Allele Frequency)≥0.05; “-”data unavailable.

**Figure 5:**
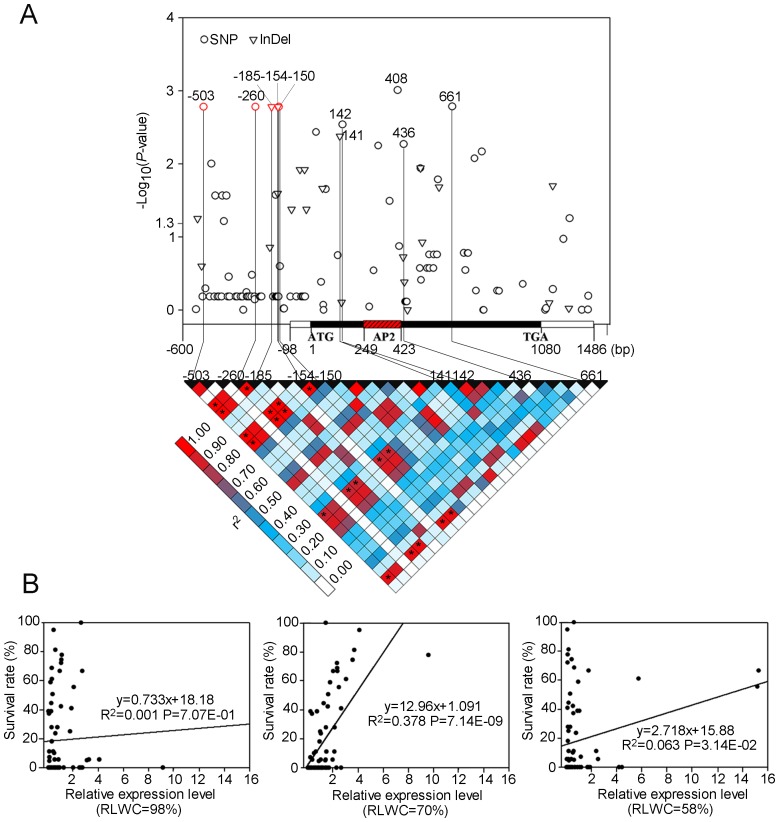
Association analysis of genetic variation *ZmDREB2.7* with maize drought tolerance. (A) Association analysis of genetic variation *ZmDREB2.7* with maize drought tolerance and the pattern of pairwise LD of DNA polymorphisms in the *ZmDREB2.7* gene. A schematic diagram of the 2.1 kb genomic fragment, including 600-bp 5′-, 406-bp 3′-UTR, and the protein coding region are presented as the x-axis. The location of the initiation codon (ATG) is marked as “+1”. The P value is shown on a −log_10_ scale. The five significant polymorphisms in the 5′-UTR and four nonsynonymous variations in the coding region are connected to their locations on the gene diagram by solid lines. “*” indicates a strong LD (r^2^≥0.8) with these five polymorphisms. (B) Correlation analysis of survival rate with the relative expression level of *ZmDREB2.7*. Drought stress was applied to the maize seedlings after the RLWC was decreased from 98% (unstressed) to 70% (moderate drought) or 58% (severe drought).

In order to fully identify the DNA polymorphism present in the *ZmDREB2.7* gene, it was re-sequenced in 105 maize inbred lines that were randomly selected from the variation panel. A 2.1 kb genomic fragment was sequenced spanning the *ZmDREB2.7* coding region and both the 5′-, and 3′-untranslated region (UTR). In total, 102 SNPs and 22 insertions or deletions (InDels) were discovered including the SNPs previously identified and reported in the RNA-seq data of 368 maize varieties (MAF≥0.05; [39]). The association of each polymorphism with drought tolerance was analyzed again using the MLM model and the pairwise linkage disequilibrium (LD) of these polymorphisms was calculated (Figure 5). Results indicated that five newly-identified polymorphisms (SNP-503, -260, -150 and InDel-185, -154), located upstream from the ATG site, were significantly associated with phenotypic variation, and were in complete LD among these materials. Additionally, three significant, nonsynonymous SNPs (SNP142, 436, 661) and a 3-bp InDel141 polymorphisms were found in the coding region. A significant synonymous variation of SNP408, located in AP2/ERF DNA-binding domain, was also detected, and it was in a strong LD with InDel141. Two of the nonsynonymous SNPs (SNP142 and SNP661) were in strong LD with the five polymorphisms in the 5′-UTR (Figure 5A). In order to determine whether or not the differences in gene expression or protein activity contribute to drought tolerance, mRNA levels of *ZmDREB2.7* under favorable, moderate and severe drought conditions were quantified in 73 randomly selected maize inbred lines. It was found that under moderate/early drought stress (RLWC = 70%), *ZmDREB2.7* gene expression level was positively correlated with increased survivability. However, no significant correlation was observed under either well-watered or severe/late drought conditions (Figure 5B). This observation indicated that an early induction of *ZmDREB2.7* gene expression in response to drought stress, rather than a basic or slow response, was important for survival of maize plants under drought stress. On the other hand, the protein transactivation assay indicated that changes in amino acids due to the four nonsynonymous mutations in the coding region did not significantly affect protein activity (Figure S3). In summary the differences in the regulation of *ZmDREB2.7* expression, but not transactivation activity of the protein, probably contributed to the natural variation in drought tolerance. Therefore, the polymorphism in the 5′-UTR of *ZmDREB2.7* may be the important functional variation conferring drought tolerance on maize seedlings.

### ZmDREB2.7 Can Bind the DRE Sequence and Enhance Plant Drought Tolerance

**Figure 6:**
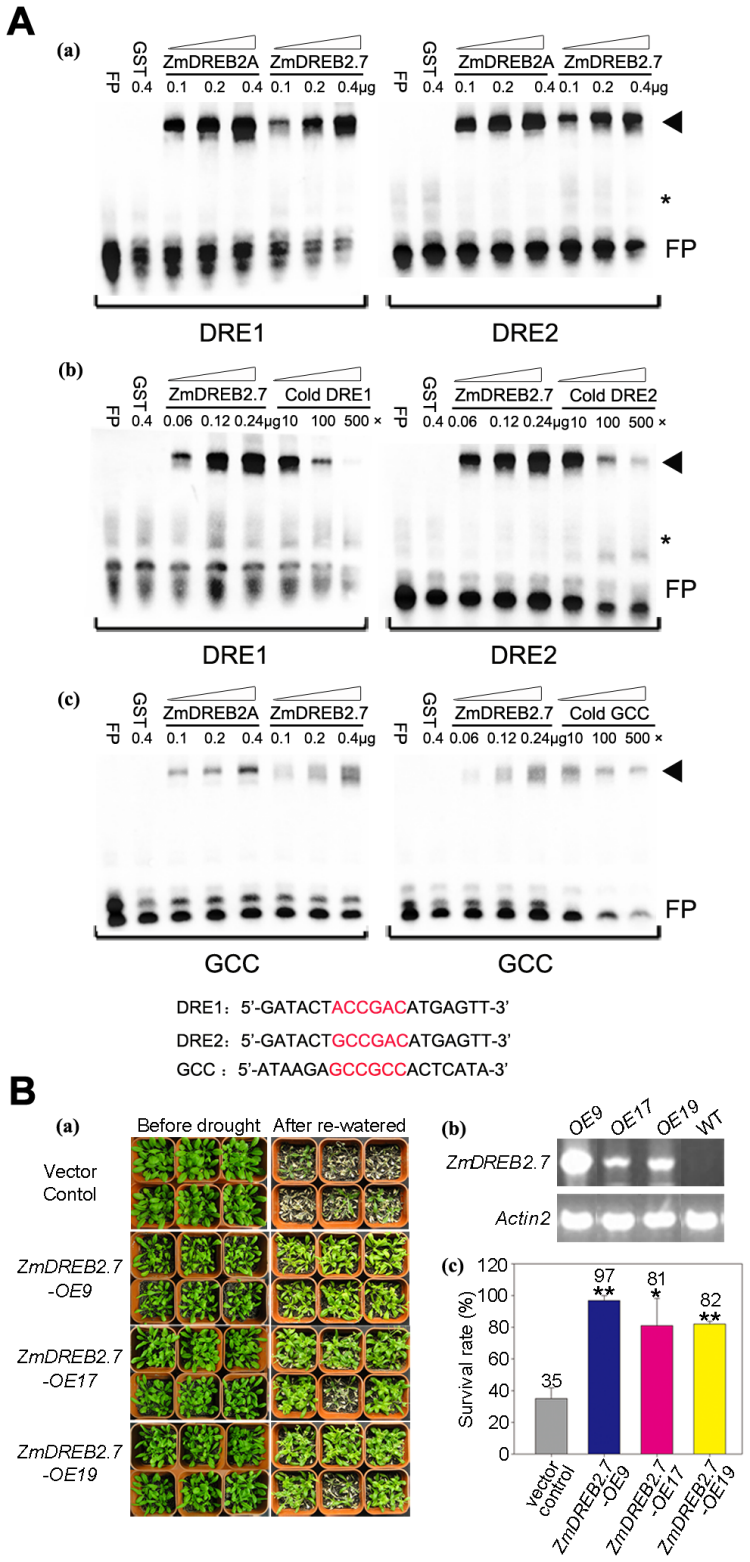
DNA binding analysis of ZmDREB2.7 and drought stress tolerance of *35S:ZmDREB2.7* transgenic *Arabidopsis* plants. (A) (a) Dosage-dependent binding of GST-ZmDREB2.7 to DRE1 and DRE2 elements. GST-ZmDREB2.1/2A was used as a positive control. (b) Competitive DNA binding assay of ZmDREB2.7 with DRE1 and DRE2 sequences. 10-, 100-, 500-fold excess amounts of the cold probe were used to compete for the binding of ZmDREB2.7 protein to the labeled probe. (c) The binding assay of GST-ZmDREB2.7 with a GCC-box. The sequences of the three kinds of DNA sequences are listed. Red letters indicate the core sequences. DNA-protein complexes are indicated by arrows; “*” indicates nonspecific bands; “FP” indicates unbound free probes. (B) (a) Drought tolerance of transgenic *35S:ZmDREB2.7 Arabidopsis* plants. Photographs were taken both before and after the drought treatment followed by 6 days rewatering. Vector-transformed plants and *ZmDREB2.7*-*OE9*, *ZmDREB2.7*-*OE17* and *ZmDREB2.7*-*OE19* transgenic plants were compared. (b) RT-PCR analysis of transcript levels in the three lines of the *35S:ZmDREB2.7* transgenic plants. (c) Statistical analysis of survival rates after the drought-stress treatment. The average survival rates and standard errors were calculated from three independent experiments. Bars with asterisks indicate lines that had significantly higher survival rates than the vector-transformed plants (t-test, *p<0.05, **p<0.01).

To determine whether or not ZmDREB2.7 is a typical DREB-type transcription factor and is able to improve plant drought stress tolerance, the protein was purified and tested for its ability to bind to the DRE sequence *in vitro*. As illustrated in Figure 6A, ZmDREB2.7-GST fusion protein could bind both typical DRE sequences, ACCGAC and GCCGAC, with a similar affinity, and the binding signal was specifically inhibited by un-labeled DNA sequences in competitive assays. When compared with ZmDREB2.1/2A protein, ZmDREB2.7 was found to possess a similar target DNA binding ability. The GCC sequence is the target DNA sequence of the ERF subgroup of TFs within the AP2/ERF superfamily and this sequence is enriched in the promoters of ethylene-responsive or biotic-stress-responsive genes [46]. TINY, one of *Arabidospsis* AP2/ERF TFs, can bind both DRE and GCC *cis*-elements equally, thus enabling crosstalk in plant biotic and abiotic stress responses [47]. When the GCC sequence was used in the binding assay, both ZmDREB2.1/2A and ZmDREB2.7 proteins displayed a very faint binding signal, indicating a low level of binding affinity. Additionally, the band intensity was only slightly weakened in the competitive assay. This indicates that the GCC sequence was not the specific DNA target site of either ZmDREB2.1/2A or ZmDREB2.7 (Figure 6A). Since overexpression of the *Arabidopsis DREB2A* gene did not result in a remarkable drought tolerant phenotype in transgenics, which is most likely a result of the instability of the ectopic expressed protein in plant cells [9], [11], we were interested to determine whether ectopic expression of the *ZmDREB2.7* gene was capable of improving stress tolerance. Transgenic *Arabidopsis* plants overexpressing the *ZmDREB2.7* gene were created and drought tolerance was observed to be significantly enhanced in all three independent transgenic lines. The survival rate of the vector-transformed control plants was 35%, while the survival of the *ZmDREB2.7* overexpressing lines ranged from 82–97% (Figure 6B). A dwarf or delayed-flowering phenotype was not observed in most of the *ZmDREB2.7*-OE lines, however, *ZmDREB2.7*-OE9 plants exhibited a slight reduction in the size of rosette leaves, which had the highest level of transgene expression (Figure 6B). Unlike *Arabidopsis* DREB2A, these data support the hypothesis that post-translational regulation might not be important for ZmDREB2.7. Protein sequence analysis indicated that ZmDREB2.7 did not contain the amino acid sequence homologous to the negative regulation domain (NRD) present in *Arabidopsis* DREB2A. Taken together, these data clearly demonstrate that *ZmDREB2.7* can specifically bind DRE sequences and overexpression of this gene can confer drought stress tolerance on transgenic *Arabidopsis*.

### Co-segregation of the Favorable/Tolerant Allele of *ZmDREB2.7* with Improved Drought Tolerance in Maize

**Figure 7:**
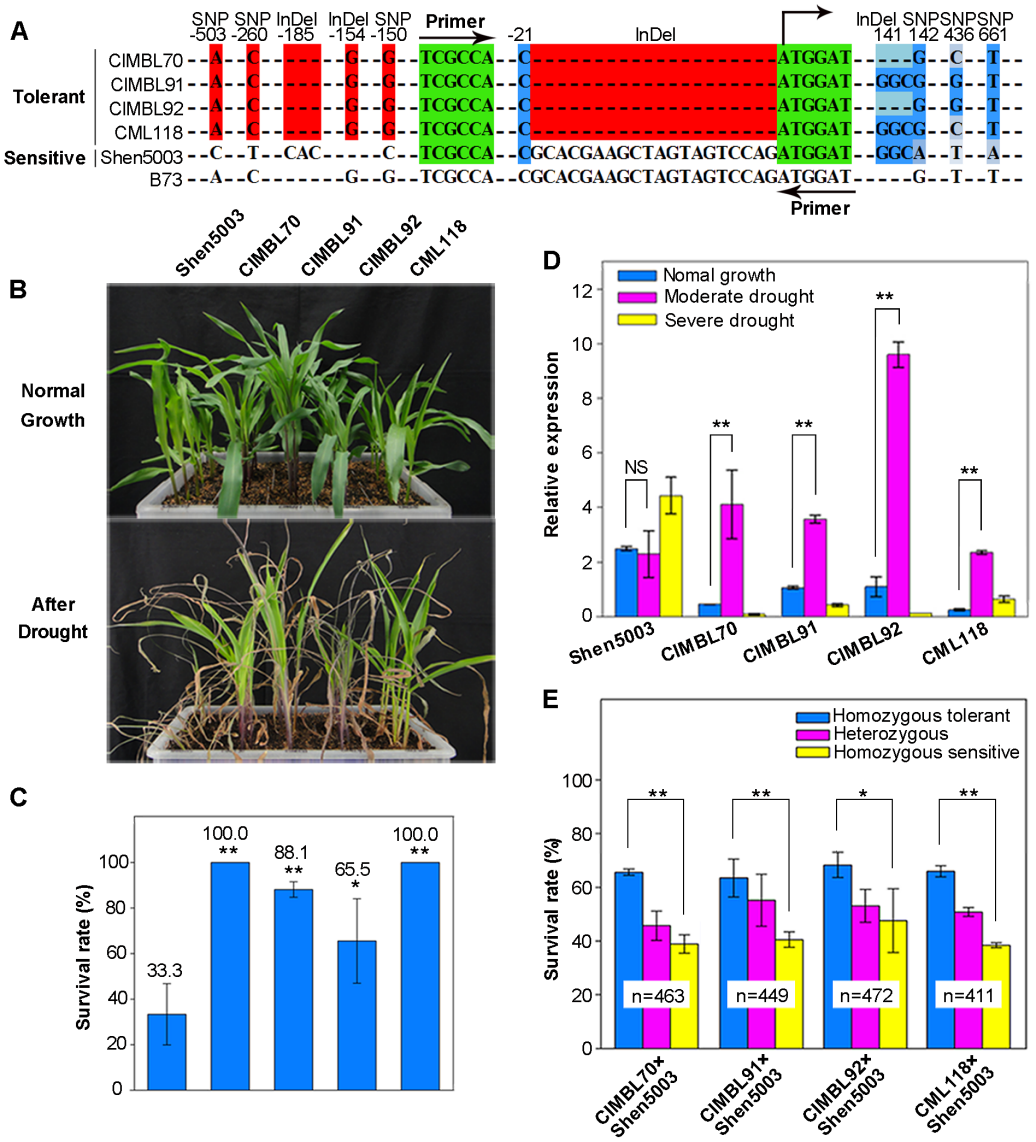
The favorable allele of *ZmDREB2.7* improves maize drought tolerance. (A) Haplotypes of *ZmDREB2.7* in CIMBL70, 91, 92, CML118, Shen5003 and B73 (as reference genome) maize genotypes. The site of the start codon (ATG) was designated as “+1”. SNP-503, SNP-260, SNP-150, InDel-185 and InDel-154 are the five DNA polymorphisms significantly associated with maize drought tolerance and are located in the 5′-UTR of *ZmDREB2.7*. The 20-bp InDel upstream of the ATG is in complete LD with the five polymorphisms in the four drought tolerant varieties. These polymorphisms are shaded in red. The location of PCR primers used for genotyping the InDel polymorphism of *ZmDREB2.7* in drought tolerant (CIMBL70, 91, 92, and CML118) and drought sensitive (Shen5003) inbred lines are indicated by arrows. The four significant nonsynonymous polymorphisms in the coding region of InDel141, SNP142, SNP436 and SNP661 are shaded in blue. (B) Phenotypic response of CIMBL70, 91, 92, CML118 and Shen5003 to drought stress. The upper panel is a photo of plants growing under favorable water conditions while the lower panel plants re-watered for 6 days after the drought stress treatment was terminated. (C) The survival rate of CIMBL70, 91, 92, CML118 and Shen5003 plants exposed to moderate and severe drought stress. Data represent the mean of triplicates (t-test, *p<0.05, **p<0.01). (D) Relative level of *ZmDREB2.7* expression in CIMBL70, 91, 92, CML118 and Shen5003 grown under normal and drought stress conditions. The drought-stress treatment reflected a decrease in RLWC from 98% (unstressed) to 70% (moderate drought), and 58% (severe drought). Data represent the mean of three biological replicates (t-test, **p<0.01). (E) The effect of the *ZmDREB2.7* favorable allele on drought tolerance in four F2 segregating populations of maize. In each population, three distinct genotypes for *ZmDREB2.7* were identified by DNA amplification: homozygous for the favorable allele, homozygous for the sensitive allele, and heterozygous for both alleles. The survival rate of the different genotypes was assessed and compared in the four populations. N indicates the number of F2 individuals tested in each population (t-test, *p<0.05, **p<0.01).

In order to compare the genetic effect of different *ZmDREB2.7* alleles on drought tolerance in maize, four drought-tolerant, inbred lines (CIMBL70, 91, 92 and CML118 were selected and crossed with a drought-sensitive variety (Shen5003) resulting in four segregating F2 populations. All four drought-tolerant lines have the same *ZmDREB2.7* allelic sequence in the 5′-UTR at five significant loci, while Shen5003 has the opposite allele at all five loci. Thus, the *ZmDREB2.7* allele in the tolerant inbred lines was considered to be the favorable/tolerant allele and the allele in Shen5003 was inferior/sensitive. The DNA polymorphisms of the five varieties at the significant loci are shown in Figure 7A. Additionally, a 20-bp InDel, located 21-bp upstream of the start codon, was found in the four drought-tolerant inbred lines. Although this polymorphism was not as significantly associated with drought tolerance in the 105 varieties as was the five loci located in the 5′-UTR, a pair of primers, surrounding the 20-bp InDel, was designed to distinguish the presence of the favorable *ZmDREB2.7* allele by PCR, due to their close physical linkage (Figure 7A). A comparison of the level of drought tolerance in the five parental materials is shown in Figures 7B. When drought stress was applied to the plants, about 33.3% Shen5003 plants survived, while survival rate of the CIMBL70, 91, 92 and CML118 was 100%, 88.1%, 65.5% and 100%, respectively (Figure 7C). Expression of *ZmDREB2.7* was significantly induced in the four tolerant genotypes in response to a moderate drought stress (RLWC = 70%) whereas, it was not significantly upregulated at all in the sensitive genotype (Figure 7D).

More than 400 individual F2 plants in each of the four F2 segregating populations were genotyped for the presence of the favorable/tolerant allele of *ZmDREB2.7* by PCRs. As expected, a Mendelian inheritance pattern was observed for the *ZmDREB2.7* favorable/tolerant allele in each of the four segregating populations. The segregation rate of homozygous tolerant, heterozygous tolerant/sensitive, and homozygous sensitive plants was approximately 1∶2∶1 (Figure S4; Table S6). The survival rates of plants carrying the three different assortments of *ZmDREB2.7* alleles were then compared after being subjected to a drought stress. As shown in Figure 7E, plants that were homozygous for the favorable/tolerant allele of *ZmDREB2.7* were more tolerant to drought stress than plants that were homozygous for the inferior/sensitive allele. Plants that were heterozygous for the favorable and inferior alleles exhibited a level of drought tolerance that was intermediate between the plants that were homozygous favorable or homozygous inferior. Co-segregation of the *ZmDREB2.7* tolerant allele with improved drought tolerance suggested the linkage of this locus with the trait in segregation populations. In maize, linkage analyses using bi-parental crosses also reported QTLs (quantitative trait loci) for drought tolerance within the chromosomal region (Chr. 1, bin 1.07) where the *ZmDREB2.7* gene located [48]–[50]. Collectively, these data further support the premise that natural variation in *ZmDREB2.7* contributes to enhanced drought tolerance in different maize varieties. Importantly, the tolerant/favorable allele of *ZmDREB2.7* represents a promising genetic resource for the development of drought-tolerant maize cultivars using traditional breeding approaches or genetic engineering.

## Discussion

Although a number of reports have indicated the important role played by *DREB*-type transcription factors in the regulation of plant response and adaptation to multiple abiotic stresses, including drought stress, little is known about the diverse functions of individual *DREB* genes and whether or not quantitative differences in the DREB response pathway may contribute to the natural variation in plant response to drought stress observed between and within a species. Answering this question can have a great practical benefit for the development of stress-tolerant crops. In the present study, we were able to utilize recent advances in maize genomic research and high-throughput sequencing technology, to systematically identify *DREB*-type genes in the maize B73 genome and determine if polymorphisms in the sequence of *DREB*-type genes were associated with drought tolerance. While much of the previous research has studied the function of an individual gene in a single genotype, the present study provides comprehensive information regarding DREB-type TF gene function in maize drought tolerance through the association analysis. Moreover, biochemical and transgenic studies further supported the natural variation in *ZmDREB2.7* gene contributed to maize drought tolerance at seedling stage.

In the present study, ten *DREB1*-type genes were identified in the maize B73 genome. This gene number is conserved among rice, maize and sorghum although it is different from the number present in *Arabidopsis* (Figure 1). Phylogenetic analysis showed that monocot DREB1 proteins cluster independently from those in *Arabidopsis* suggesting a potential functional diversity between dicot and monocot plants. In *Arabidopsis*, DREB1 proteins are the major TFs involved in plant cold stress response. Transcriptomic and metabolomic studies in plants overexpressing *DREB1A* genes indicated that genes for starch degrading enzymes and sugar alcohol synthases, as well as the resultant metabolites, were upregulated just as they are in plants exposed to low temperature. These data suggest that a re-allocation of energy and/or an osmotic adjustment is involved in stress adaptation [51]. Similar molecular mechanisms and physiological responses, induced and regulated by *DREB1* genes, appears to occur in monocots [17], [52], [53]. The reason why monocot plants possess an increased number of *DREB1* genes, compared to Arabidopsis, is unknown. We found that the majority of *ZmDREB1* genes were expressed at high levels in roots under normal growing conditions (Figure 3A). Whether they play a role in maize root development will require further investigation. The presence and organization of specific conserved motifs in the ZmDREB1 proteins exhibited a high degree of similarity amongst the various members while transactivation activity differed from low to high levels indicating that other residues outside of the conserved motifs may play an important role in transactivation activity (Figure 4).

In relative comparison with DREB1s, DREB2 proteins are more phylogenetically diversified in the four species examined. Although ten *ZmDREB*2s genes were predicted based on a BLAST of the B73 maize genome, only eight genes could be cloned. An analysis of gene structure indicated that the two genes were probably pseudogenes, especially since no transcripts were ever identified. The two putative, pseudogenes may have originated and subsequently become dysfunctional during evolution of the maize genome (Figure 1). Only two clades in the *DREB2* subgroup, each containing a single *ZmDREB2* member (either ZmDREB2.3/ZmABI4 or ZmDREB2A/2.1), were evolutionarily conserved across the four species analyzed (corn, rice, sorghum, and *Arabidopsis*) (Figure 1). In *Arabidopsis*, *ABI4* encodes a TF involved in ABA signaling, seed maturation and lateral root formation [54]–[56]. Rice homologs of this gene were also classified in the DREB2 subgroup based on protein sequence similarity [19], thus *ZmDREB2.3/ZmABI4* was also closely examined in our study. Similar to the expression pattern of this gene in *Arabidopsis*, *ZmDREB2.3/ZmABI4* was found to be highly expressed in germinating seeds and embryos (Figure 3A). Notably, the expression of this gene was clearly inducible in both leaves and roots under drought stress, indicating a possible role for this gene in the stress response (Figure 3B). Supporting this premise is the fact that in the association analysis, the genetic variation of *ZmDREB2.3/ZmABI4* was found to linked to the phenotypic variation in drought tolerance, although its association was less significant than *ZmDREB2.7* (Table 1). In all likelihood, genetic polymorphisms in both *ZmDREB2.7* and *ZmDREB2.3/ZmABI4* contribute variations in maize drought tolerance. Additional copies of *ZmDREB2.4, 2.5* and *2.6* and *ZmDREB2.7*and *2.8* were only found in maize, enlarging the number of *DREB2*-type genes in this species (Figure 1). The chromosomal segments containing these genes were also found to be duplicated, supporting the concept that the maize genome may have arisen from an ancestral allotetraploid, half of which shares a common ancestor with sorghum, which in turn probably represents a lineage split in rice [57], [58]. The biological significance of *DREB2* gene duplications in the maize genome remains to be determined.

Previous efforts have been made to explore the association of *DREB1s* or *DREB2s* with plant stress tolerance in a number of plants, including *Arabidopsis*, common bean, and foxtail millet [59]–[61]. In these studies, a few homologous *DREB* genes were investigated in a small number of varieties and predictive conclusions were proposed. Recent advances in maize genomics, and the availability of genetically diverse collections composed of hundreds of varieties enabled us to systematically undertake an association analysis in maize. We evaluated the drought stress tolerance of seedlings to a severe water stress for each genotype of a maize population consisting of 368 varieties from tropical and temperate regions. The size and genetic diversity of this population makes it ideal for use in a complex trait association studies [39], [42]. RNA-seq data from this population identified 525,105 high quality SNPs, present in more than 25,000 maize genes [39]. This enabled us to study the association of 14 *ZmDREB* genes with drought stress tolerance. After controlling for population structure and cryptic relatedness, both of which may cause spurious associations, genetic polymorphism in *ZmDREB2.7*, among all of the 14 *ZmDREB* genes analyzed, was identified to be the most significantly associated with phenotypic variation in drought tolerance (P<0.001, Table 1) using the MLM model. When population structure was controlled by the use of a Q matrix (calculated by STRUCTURE), similar results were obtained. Further sequencing and association analyses identified five DNA polymorphisms in the 5′-UTR of *ZmDREB2.7* that were associated to drought tolerance variation (Figure 5A). In the B73 genotype of maize, *ZmDREB2.7* expression was detected at a low level in various tissues and was slightly induced in leaves and roots by drought stress. However, the ZmDREB2.7 protein possessed a high level of transactivation activity compared with other ZmDREB proteins (Figure 3 and 4). In support of the association study, it was found that the expression level of *ZmDREB2.7*, but not the activity of the protein, was correlated with drought tolerance among different maize varieties (Figure 5B). Moreover, ZmDREB2.7 protein-DNA binding analysis *in vitro* and analysis of the effect of *ZmDREB2.7* overexpression in transgenic *Arabidopsis* demonstrated that *ZmDREB2.7* can function as a typical DREB-type TF and improve drought tolerance (Figure 6).

The majority of *ZmDREB1*s, as well as some *ZmDREB2*s such as the previously identified *ZmDREB1.1/1A* and *ZmDREB2.1/2A*, are highly induced in leaves and/or roots in response to drought stress in the B73 in bred line. However, genetic polymorphisms in these genes were not as significantly associated with drought stress as those of *ZmDREB2.7*. We suggest that some genes may be essential to stress response in all the maize varieties and that significant genetic variation in those genes would either result in a lethal phenotypic defects or an undetectable effect, the latter being due to functional compensation by other redundant genes. Therefore, we could not detect a significant association of these genes. In spite of a large degree of variation in the survival rates among the 368 varieties after being subjected to drought stress, basic stress responses (e.g. stress-related gene induction, stomatal closure) could still be observed even in the most sensitive genotypes, indicating that the central or basic response was still conserved in this variation panel. Gene transfer technology could be used to modulate the expression of a highly stress-inducible gene and thus improve tolerance to stress. This approach was evidenced by the creation of *ZmDREB1.1/1A* and *ZmDREB2.1/2A* overexpressors, however, their expressions or activity require optimization to avoid negative effects on plant growth and yield [17], [18]. Additionally, four *ZmDREB* genes in our current dataset were not polymorphic. Therefore, their association with phenotypic variation in drought stress could not be estimated. Whether or not polymorphisms in these genes are important for drought tolerance remains undetermined. The fact that genetic variation in the five loci upstream the start codon of *ZmDREB2.7* could only explain 6.68% of the variation in drought tolerance among the maize population is consistent with the notion that drought tolerance is a complex trait underlined by a number of contributing genes (Table S7). In the future, a whole genome scale GWAS for maize drought tolerance at the seedling stage will be further investigated. It is anticipated that this complex analysis will provide an overview of the genetic contribution to this trait.

DREB2A protein was reported to prefer ACCGAC to GCCGAC as a target binding site, although both sequences represent a typical DRE *cis*-element [9]. We found that ZmDREB2.7 protein can equally bind both DRE target sequences *in vitro*, indicating that differences in DNA-binding-preference may exist between DREB2A and ZmDREB2.7 proteins. ZmDREB2.7 did not interact with the GCCGCC sequence in a specific manner, suggesting that this protein is mainly involved in drought stress rather than ethylene or biotic stress response (Figure 6A). Together with its high level transactivation activity, it is suggested that, in response to drought stress, ZmDREB2.7 protein can bind and activate the promoter of downstream stress-responsive genes. Although the DNA binding preference of ZmDREB2.1/2A and ZmDREB2.7 is generally similar, at a low protein concentration ZmDREB2.1/2A showed a higher affinity for the DRE sequences than ZmDREB2.7 (Figure 6A). Transgenic plants overexpressing *ZmDREB2.7* exhibited improved plant drought stress tolerance which strongly supports the contention that regulation of gene expression was an important function for this gene. Previous reports indicated that transgenic plants constitutively overexpressing *DREB2A-CA* or *ZmDREB2.1/2A* gene exhibited a dwarf phenotype in addition to enhanced drought tolerance [9], [18]. In the present study, significant growth retardation was not observed in most of the *ZmDREB2.7* transgenic plants, except a mild phenotype of *ZmDREB2.7*-OE9 plants, which had the highest level of transgene expression (Figure 6B). Probably, different *DREB* genes may differentially affect plant growth and development in *Arabidopsis* due to their different binding affinity to target DNA sequences.

The function of DREB TFs is to bind DRE sequences present in the promoter region of many stress-inducible genes and transactivate gene expression, the gene products of which may protect plants from stress impairment [1]. Thus, an early and quick response to an environmental stress signal is important for the proper function of a TF gene. This can be accomplished either by a rapid induction of gene expression in response to an environmental stimulus or by quick modulation of transactivation activity of the protein coded by the TF. In our study, genetic polymorphisms in the 5′-UTR of *ZmDREB2.7* were associated with variation in maize drought tolerance. Furthermore, differences in *ZmDREB2.7* gene expression in response to a moderate drought stress, but not severe drought or normal growth conditions, were correlated with plant survival among different maize varieties (Figure 5). It suggested induction of *ZmDREB2.7* expression in early drought stress was important for plant survival in stress, which coincided with its function as a TF to activate downstream stress-responsive gene expression. The quicker induction of *ZmDREB2.7* expression in the tolerant genotype of CIMBL70, 91, 92 and CML118 than in the sensitive genotype of Shen5003 was consistently observed (Figure 7D). We further analyzed the *ZmDREB2.7* gene expression data in approximately seventy maize inbred lines based on tolerant or sensitive genotypes of *ZmDREB2.7*, under well-watered, early and late drought stress conditions. The results demonstrated that, on average, the materials carrying the tolerant allele of *ZmDREB2.7* had a significantly higher expression level than those carrying the sensitive allele in response to early drought stress (Figure S5). Moreover, we found that among the 105 randomly selected varieties, the subpopulation consisting of tropical inbred lines had the highest frequency of the favorable allele of *ZmDREB2.7*, which is consistent with the observed higher level of drought tolerance of this subpopulation than the others (Figure S6). We searched the database of Plant Cis-acting Regulatory DNA Elements (http://www.dna.affrc.go.jp/PLACE/signalscan.html) to identify putative *cis*-elements in the obtained 570-bp sequences upstream from the start codon amongst the 105 genotypes. Among the most significant 5 loci and the 20-bp InDel polymorphism, only InDel-154 brings about an additional W-box in the promoter of Shen5003, which is possibly a WRKY TF recognition site. At the present time it is not known whether this difference results in alteration of gene expression in response to drought stress. Four nonsynonymous polymorphisms in the *ZmDREB2.7* coding region (outside of the DNA-binding domain) were detected to be significantly associated with drought tolerance (Figure 5A) but the proteins encoded by the different haplotypes displayed similar levels of transactivation activity (Figure S3), indicating that they were probably not causal variations that affected the function of the coded protein.

In the present research we found that: (1) three SNPs and two InDels, upstream of the start cordon of *ZmDREB2.7* were significantly associated with phenotypic variation in drought tolerance (Figure 5A); (2) nonsynonymous mutations in the protein coding region did not greatly affect transactivation activity of the coded protein (Figure S3); (3) consistent with the TF function of ZmDREB2.7, a rapid induction of *ZmDREB2.7* gene expression in response to a moderate drought stress was important in conferring plant drought-stress tolerance (Figure 5B); (4) overexpressing *ZmDREB2.7* can improve drought stress tolerance in transgenic plants (Figure 6B); (5) the favorable allele of *ZmDREB2.7* could effectively enhance plant drought tolerance in four distinct genetic backgrounds compared to the inferior allele (Figure 7E). We conclude that naturally occurring polymorphisms in *ZmDREB2.7* contribute to drought stress tolerance of maize seedlings and that the polymorphisms in the gene promoter region were the functional variations responsible for the observed variations in gene expression and plant drought tolerance. The identified beneficial allele may be of more practical use rather than as a transgene since gene expression may have been optimized during evolution and/or natural selection *in planta*. Increasing gene function through ectopic gene expression usually results in pleiotropic effects, such as growth and/or developmental defects, which is especially true for overexpession of the regulatory gene. *ZmDREB2.7* and its favorable allele may be a valuable genetic resource for improving maize drought tolerance either as a genetic marker in marker assisted breeding or in transgenic approaches.

## Materials and Methods

### Phylogenetic Tree Construction

Full-length amino acid sequences of 66 *DREB1s* and *DREB2s* identified in maize, rice, *Arabidopsis* and sorghum were aligned using the Clustal X 1.83 program with default pairwise and multiple alignment parameters. The phylogenetic tree was constructed based on this alignment result using the neighbor joining (NJ) method in MEGA version 5 (http://www.megasoftware.net/) with the following parameters: Poisson correction, pairwise deletion, uniform rates and bootstrap (1000 replicates). The ZmDREB proteins were named sequentially according to their placement in the phylogenetic tree.

### Plant Growth and Drought Treatment

Maize seeds were surface-sterilized in 1‰ (v/v) Topsin-M (Rotam Crop Sciences Ltd.) for 10 min, then washed in deionized water and germinated on wet filter paper at 28°C for 3 days. The germinated seeds were either transplanted to enriched soil (turf to vermiculite in a ratio of 1∶1) or placed in a nutrient solution (0.75 mM K_2_SO4, 0.1 mM KCl, 0.25 mM KH_2_PO_4_, 0.65 mM MgSO_4_, 0.1 mM EDTA-Fe, 2 mM Ca(NO3)_2_, 1.0 µM MnSO4, 1.0 µM ZnSO_4_, 0.1 µM CuSO_4_, 0.005 µM (NH4)_6_Mo_7_O_24_ for hydroponic cultivation [62]. Drought treatment was applied to the soil-grown plants at the 3-leaf seedling stage by withholding water. Leaf samples for gene expression analyses were collected when relative leaf water content (RLWC) decreased to 98%, 70%, 60% and 58%, which reflected different levels of drought stress. For root samples, the hydroponic cultured seedlings at a corresponding developmental stage were placed on a clean bench and subjected to dehydration (28°C, relative humidity of 40∼60%). Samples were exposed for 0, 5, 10 and 24 hours, the time point of which was determined by measuring the RLWC, corresponding to the drought-treated leaf samples, which were approximately 98%, 70%, 60% and 58%, respectively.

### Expression Profile of *ZmDREB* Genes

Expression patterns of 18 *ZmDREB*s in different maize tissues were analyzed using the genome-wide gene expression atlas of the inbred B73 line of maize that was reported previously [41]. Expression data for the 15 tissues were combined from 60 growth stages. Normalized expression values of each gene in different tissues were averaged. The gene expression level was presented as a log value. The responsiveness of each *ZmDREB* gene to drought stress was analyzed by qRT-PCR and the expression of *ZmUbi-2* (UniProtKB/TrEMBL; ACC:Q42415) was used as an internal control. Total RNA was isolated using TRIZOL reagent (Biotopped) from no less than 3 seedlings. In order to eliminate genomic contamination, total RNA was treated with RNase-free DNAse (Takara). The concentration of total RNA was determined using a Nanodrop1000 (Thermo Scientific product, USA). In order to confirm RNA integrity and quantity, 5 µg of total RNA from each sample was run on a 0.8% agarose gel. Recombinant M-MLV reverse transcriptase and 1 µg of total RNA mixed with 1 µg Oligo (dT) 23 (Promega) were used to synthesize the cDNAs.

### Transactivation Activity Assay

Eighteen *ZmDREB* genes were individually cloned into the pBluescript II KS+ vector from the maize B73 inbred line. After sequence analysis, the *ZmDREB* genes were transferred to pGBKT7 for evaluation of transactivation activity in the AH109 yeast strain. The cell concentration of yeast transformants was adjusted to an OD_600_ of 0.1, the yeast cells were then dropped on SD/-T, SD/-T-H, SD/-T-H-A and SD/-T-H-A plates containing various concentrations of 3-AT to compare their ability to grow. The plates were incubated at 30°C for 2–5 days before photographing.

### Preparation of ZmDREB2.7-GST Fusion Protein and Gel Mobility Shift Assay

DNA fragments of *ZmDREB2.7* and *ZmDREB2.1/2A* encoding the AP2/ERF DNA-binding domain were cloned into the *Eco*R I-*Sma* I sites of pGEX4T-1 vector and transformed into the *E. coli* strain of Rosseta pLys. The primers used for amplification of the fragments of *ZmDREB2.7* and *ZmDREB2.1/2A* were 5′-TTGAATTCATGGATCGGGTGCCG-3′ and 5′-TCACTGCAGGTTTAGGCGAGC-3′, 5′-GGGAATTCATGACGCTGGATCAG-3′ and 5′-TCAGGGGAAGTTAGTCCGTGC-3′, respectively. The GST fusion proteins were extracted and purified using the GST-Sefinose resin as described in [6]. Gel mobility shift assays were performed according to the instructions provided with the LightShift Chemiluminescent EMSA Kit (Thermo Scientific). The DRE and GCC-box sequences which were end-labeled with biotin were 5′biotin-TTGATACTA/GCCGACATGAGTTGATACTA/GCCGACATGAGT-3′ and 5′biotin-ACTCATGTCGGTAGTATCAACTCATGTCGGTAGTATCAA-3′, respectively.

### Drought Tolerance Evaluation of the Maize Natural Variation Panel

A natural variation panel of maize consisting of 368 maize inbred lines [39] was planted in a cultivation pool (6×1.4×0.22 m, length×width×depth) in which 5-ton of loam were uniformly mixed with 0.25-ton of chicken manure. Each pool was divided into 250 plots. Twelve plants were grown of each genotype in each plot. Watering was withheld when the seedlings had three true leaves. The time point for rehydration was determined by the characterization of drought resistance among all the genotypes, e.g. the wilting rate. Typically, this occurred seven days after the soil relative water content had decreased to nearly 0%. Watering was then resumed in order to recover the surviving plants. After rehydration for 6 days, the survival rate of each genotype was assessed. The drought phenotypic data were obtained from independent replicated experiments.

### Association Analysis of *ZmDREB* Genes with Variations in Drought Tolerance

Principle components of the association panel were calculated by EIGENSTRAT [63] using the high-quality 525,105 SNP data [39] with MAF≥0.05. The first two dimensions were used in the principle components (PCA) to estimate the population structure, which could explain 11.01% of the phenotypic variation and was comparable to that calculated by STRUCTURE. The analysis was completed by the lm function in R program (http://www.R-project.org). Single-maker association analysis was done first to filter markers that had no relationship with the trait (p≥0.995). After that, 1,822 SNP markers distributed on each chromosome were chosen to estimate the kinship coefficient (K) by SPAGeDi [64]. GLM method was applied to perform single-maker analysis. A mixed linear method [43], [44], taking account of both the kinship coefficients and the population structure (PCA+K), was applied to identify the positive association of DNA polymorphisms with drought tolerance.

### *Arabidopsis* Transformation and Drought Tolerance Assay of Transgenic Plants

The coding region of the *ZmDREB2.7* cDNA of the maize B73 inbred line (1080 bp), digested with *Sma* I and *Sal* I (Takara), was inserted into the pGreen0029-35S-Ω vector [11]. The constructed plasmid carrying the desired gene was transformed into *Agrobacterium tumefaciens* GV3101+pSoup. *Arabidopsis thaliana* ecotype Col-0 was transformed as described previously [6]. Using kanamycin-based selection, several independent T2 transgenic lines were obtained, and expression of *ZmDREB2.7* transgene was confirmed in these lines by RT-PCR. Three independent overexpression lines *ZmDREB2*.7-*OE9*, *ZmDREB2*.7-*OE17* and *ZmDREB2*.7-*OE19* were selected based on the level of transgene expression and subjected to further analyses. Seven-day-old plants were transferred into pots containing 100 g soil/pot. Thirty two-day-old plants growing under favorable water conditions were exposed to drought stress. Water was withheld from the plants for 14 days. Watering was then resumed to allow plants to recover. Six days later, the number of surviving plants was recorded. At least 30 plants of each line were compared with WT in each test and statistical data were obtained from three independent experiments.

### *ZmDREB2.7* Gene Sequence and Association Analysis with Drought Tolerance among 105 Maize Genotypes

In order to amplify the full length *ZmDREB2.7* gene, including 5′ and 3′-UTR sequence, in different maize inbred lines, three pairs of primers were synthesized using the B73 genome sequence as a reference (MaizeGDB release 5b.60, http://www.maizegdb.org/). All primers were designed using Primer Express 3.0 (Table S7). All of the obtained sequences were aligned using MEGA version 5 (http://www.megasoftware.net/). Nucleotide polymorphisms, including SNPs and InDels, were identified (MAF≥0.05). The significance of each DNA polymorphism associated with maize drought tolerance was calculated using the above-mentioned PCA+K model.

### *ZmDREB2.7* Favorable Allele Detection in Segregating Populations of Maize

Four segregating populations (CIMBL70×Shen5003, CIMBL91×Shen5003, CIMBL92×Shen5003, and CML118×Shen5003) were generated. Maize seedlings were grown in enriched soil (turf to vermiculite in a ratio of 1∶1) in plastic boxes (0.70×0.50×0.18 m, length×width×depth). Each box contained 144 seedlings. Three independent replications were performed in a greenhouse using 16-h-light/8-h-dark, 28/22°C and a RH of 60%, to obtain the statistical data. A section of the cotyledons of 10-day-old plants were collected for *ZmDREB2.7* genotyping. Subsequently, drought stress was applied to the plants by withholding water. When soil relative water content decreased from 40% to 0% and wilting and death of the seedlings were visible, plants were rewatered in order to identify the surviving plants. The survival rate of each genotype was recorded. Three replications were carried out for statistical analysis. All the PCR primers used in this research were listed in Table S8.

## Supporting Information

Figure S1 Phenotypic analyses of drought tolerance at seedling stage in a natural variation population consisting of 368 maize varieties. (A) The drought tolerance of each inbred line was assessed based on the survival rate of seedlings exposed to a severe drought stress. The entire population was divided into ten groups (x-axis) and the number of varieties in each group is shown on the y-axis. (B) Variation in drought tolerance within and among subpopulations of maize. Division of the population into subpopulations (MIXED, NSS, SS, and TST) was according to Yang et al., 2011 [42] where TST = tropical or subtropical varieties; NSS = temperate varieties; SS = B73 derivatives and MIXE = varieties with no clear identity. Several highly drought tolerant outliers were identified in each subpopulation.
(TIF)

Figure S2 Quantile-quantile plot for the association of the 250 SNPs of 14 *ZmDREB* genes with maize drought tolerance. The gray line is the predicted distribution of each polymorphism under the null association. The black, blue and red dot-lines are observed distributions of GLM, PCA, PCA+K models for survival rate under drought conditions. Under the assumption that there are few true marker associations, the observed P values are expected to nearly follow the expected P values. Deviations from the expectation demonstrate that the statistical analysis may represent spurious associations [42].
(TIF)

Figure S3 The transactivation activity of ZmDREB2.7 proteins encoded by different haplotypes identified in drought tolerant and sensitive maize varieties. (A) The name of different maize inbred lines and their haplotypes at the four nonsynonymous significant sites in the coding region. (B) Yeast strain AH109 transformed with a vector carrying the *ZmDREB2.7* gene which was cloned from CIMBL70, 91, 92, CML118, Shen5003 and B73 inbred lines. Cultures of transformed yeast cells were diluted and placed on agar culture plates containing a -tryptophan (-T) synthetic dropout (SD) medium (SD/-T), -tryptophan-histidine (SD/-T-H) medium, or -tryptophan-histidine-adenine (SD/-T-H-A) medium. The (SD/-T-H-A) medium was amended with different concentrations of 3-aminotriazole (3-AT).
(TIF)

Figure S4 Genotyping of F2 individuals from the four segregating populations. Examples of PCR amplifications of DNA from F2 individuals and their parents of the four F2 populations. PCR amplification utilized primers surrounding the 20-bp InDel polymorphism upstream from the start codon of *ZmDREB2.7*. The size of the DNA band from Shen5003 was 66-bp and that from CIMBL70, 91, 92 and CML118 was 46-bp. Gel electrophoresis utilizing 3% agar was used to analyze fragments obtained by PCR amplification.
(TIF)

Figure S5 Comparison of *ZmDREB2.7* gene expression level between maize inbred lines carrying a *ZmDREB2.7* drought tolerant or sensitive allele. The allelic grouping was based on five significant polymorphisms in the 5′-UTR. “T” indicates the tolerant allele, while “S” indicates the sensitive allele. Drought stress was applied to the maize seedlings after the RLWC was decreased from 98% (unstressed) to 70% (moderate drought) or 58% (severe drought). A one-way ANOVA using the lm function in R program (http://www.R-project.org) was applied to analyze the statistical differences of relative gene expression levels in maize seedlings.
(TIF)

Figure S6 Frequency of the favorable allele of *ZmDREB2.7* among different subpopulations. The sequences corresponding to the 5′-UTR of *ZmDREB2.7* from 105 randomly selected inbred lines were analyzed. Division of the population into subpopulations (MIXED, NSS, SS, and TST) was according to Yang et al., 2011 [42] where TST = tropical or subtropical varieties; NSS = temperate varieties; SS = B73 derivatives and MIXE = varieties with no clear identity.
(TIF)

Table S1 Two hundred and ten AP2/ERF super family maize genes and their corresponding protein sequences. The gene accession number and the protein sequences were based on the maize genome sequence database (version 5b.60, http://www.maizegdb.org/). The full-length of the protein sequences were analyzed and classified into different subfamilies based on the sequence homology of the AP2/ERF DNA-binding domains. A: DREB (dehydration-responsive element-binding protein) subfamily; B: ERF (ethylene-responsive factor) subfamily; WLG = a WLG motif within a protein.
(XLSX)

Table S2 Amino acid sequences of the AP2/ERF DNA-binding domains encoded by 163 maize genes. One hundred and sixty-three genes were classified into two groups (A and B). The A group contained 65 genes that encode DREB/CBF-like proteins while the B group contained 98 genes encoding ERF-like proteins. Each group was further divided into 6 groups (A1-6 and B1-6) based on the sequence similarity of the amino acid sequence of the AP2/ERF DNA-binding domain. A-1 and A-2 groups contain the genes encoding canonical DREB1 and DREB2-type TFs.
(XLSX)

Table S3 Genes and corresponding protein sequences used for the phylogenetic tree construction. The phylogenetic tree was constructed using the full-length of the protein sequence of 66 canonical DREB1 and DREB2 genes from Arabidopsis, rice, maize and sorghum. Two groups were established as DREB1 and DREB2. The DREB1 (A1-labeled) group contains 36 proteins, and the DREB2 group (A2-labeled) contains 30 proteins. The gene names for AtDREB1s, OsDREB1s and OsDREB2s were according to Sakuma et al., 2002 [7], Mao et al., 2012 [65], and Matsukura et al., 2010 [19].
(XLSX)

Table S4 Genomic syntenic blocks among maize, rice and sorghum genomes. The gene colinearity data were collected from the Plant Genome Duplication Database (PGDD, http://chibba.agtec.uga.edu/duplication/). The maize, sorghum, and rice genome was abbreviated as Zm, Sb, and Os, respectively. The chromosomes of different species were abbreviated as “chr” plus Arabic numbers. The physical initiation position of each homozygous *DREB* gene and the resident genomic block in different genomes are labeled as “Zm_start, Os_start, Sb_start, Zm_block_start, Os_block_start and Sb_block_start”. The ending positions are labeled as “Zm_block_end, Os_block_end and Sb_block_end”.
(XLS)

Table S5 Survival rate for maize genotypes in the maize variation panel exposed to severe drought conditions. The 368 inbred lines in this natural variation panel can be classified into 3 subgroups, on the basis of their survival rate (SR), tolerant (SR≥40%), moderately tolerant (40%>SR≥10%), and sensitive (SR = 0%). Means for survival rate of seedlings exposed to severe drought conditions were calculated from independent replicated experiments. “N.D.” indicates data unavailable. The classification of subpopulations was based on Yang et al., 2011 [42].
(XLSX)

Table S6 Analysis of the genetic effect of the favorable allele of *ZmDREB2.7* on drought tolerance in four F2 populations. The *ZmDREB2.7* locus segregated as 3 genotypes among all the F2 progenies: homozygous for the favorable allele, heterozygous for both alleles, and homozygous for the sensitive allele. The haplotypes formed by the five polymorphisms (3 SNPs and 2 InDels) in the 5′-UTR of *ZmDREB2.7* were based on the genotypic analyses of the 20-bp InDel using PCR. A one-way ANOVA was applied to analyze the statistical difference in the survival rate among the “a, b, c” genotypes. “P<0.05; “A, B, C” indicated P<0.01. “N” was the number of F2 individuals tested.
(XLSX)

Table S7 Percentage of phenotypic variation explained by the favorable allele of *ZmDREB2.7*. The phenotypic variation for plant drought tolerance attributed to each polymorphism was calculated using MLM, as described in Materials and Methods. The favorable allele at each locus is underlined.
(XLS)

Table S8 Gene primers used for gene cloning, qRT-PCR, and sequencing analyses. Primer names were based on the gene name and the intended use of the primer. The numbers in brackets annotate the location of a primer within the corresponding gene. The location of the initiation codon (ATG) was considered as +1.
(XLSX)
