# Supplementary figures and images for: Genome-Wide Analysis of ZmDREB Genes and Their Association with Natural Variation in Drought Tolerance at Seedling Stage of Zea mays L
Source: PLoS Genet. 2013 Sep 26;9(9):e1003790. doi: 10.1371/journal.pgen.1003790 (PMC3784558; doi:10.1371/journal.pgen.1003790)

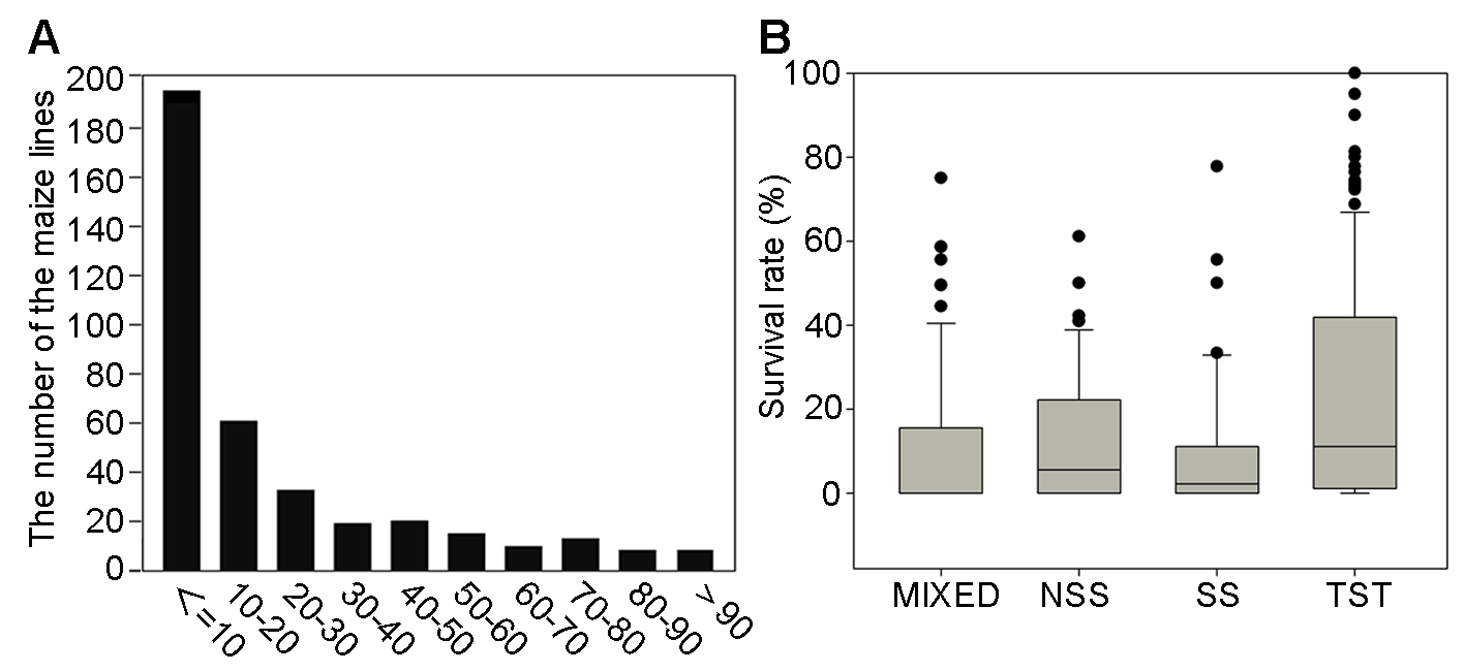

Supplement: Figure S1 — Phenotypic analyses of drought tolerance at seedling stage in a natural variation population consisting of 368 maize varieties. (A) The drought tolerance of each inbred line was assessed based on the survival rate of seedlings exposed to a severe drought stress. The entire population was divided into ten groups (x-axis) and the number of varieties in each group is shown on the y-axis. (B) Variation in drought tolerance within and among subpopulations of maize. Division of the population into subpopulations (MIXED, NSS, SS, and TST) was according to Yang et al., 2011 [42] where TST = tropical or subtropical varieties; NSS = temperate varieties; SS = B73 derivatives and MIXE = varieties with no clear identity. Several highly drought tolerant outliers were identified in each subpopulation. (TIF) [file pgen.1003790.s001.tif]

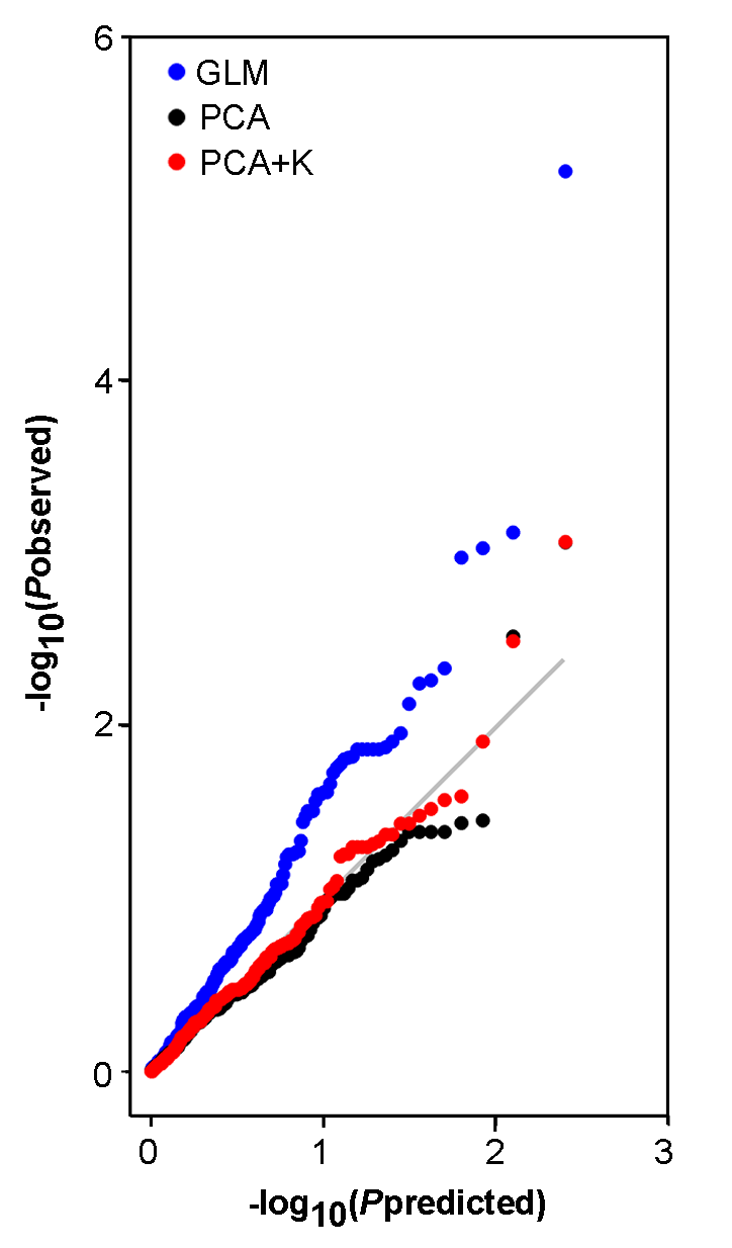

Supplement: Figure S2 — Quantile-quantile plot for the association of the 250 SNPs of 14 ZmDREB genes with maize drought tolerance. The gray line is the predicted distribution of each polymorphism under the null association. The black, blue and red dot-lines are observed distributions of GLM, PCA, PCA+K models for survival rate under drought conditions. Under the assumption that there are few true marker associations, the observed P values are expected to nearly follow the expected P values. Deviations from the expectation demonstrate that the statistical analysis may represent spurious associations [42]. (TIF) [file pgen.1003790.s002.tif]

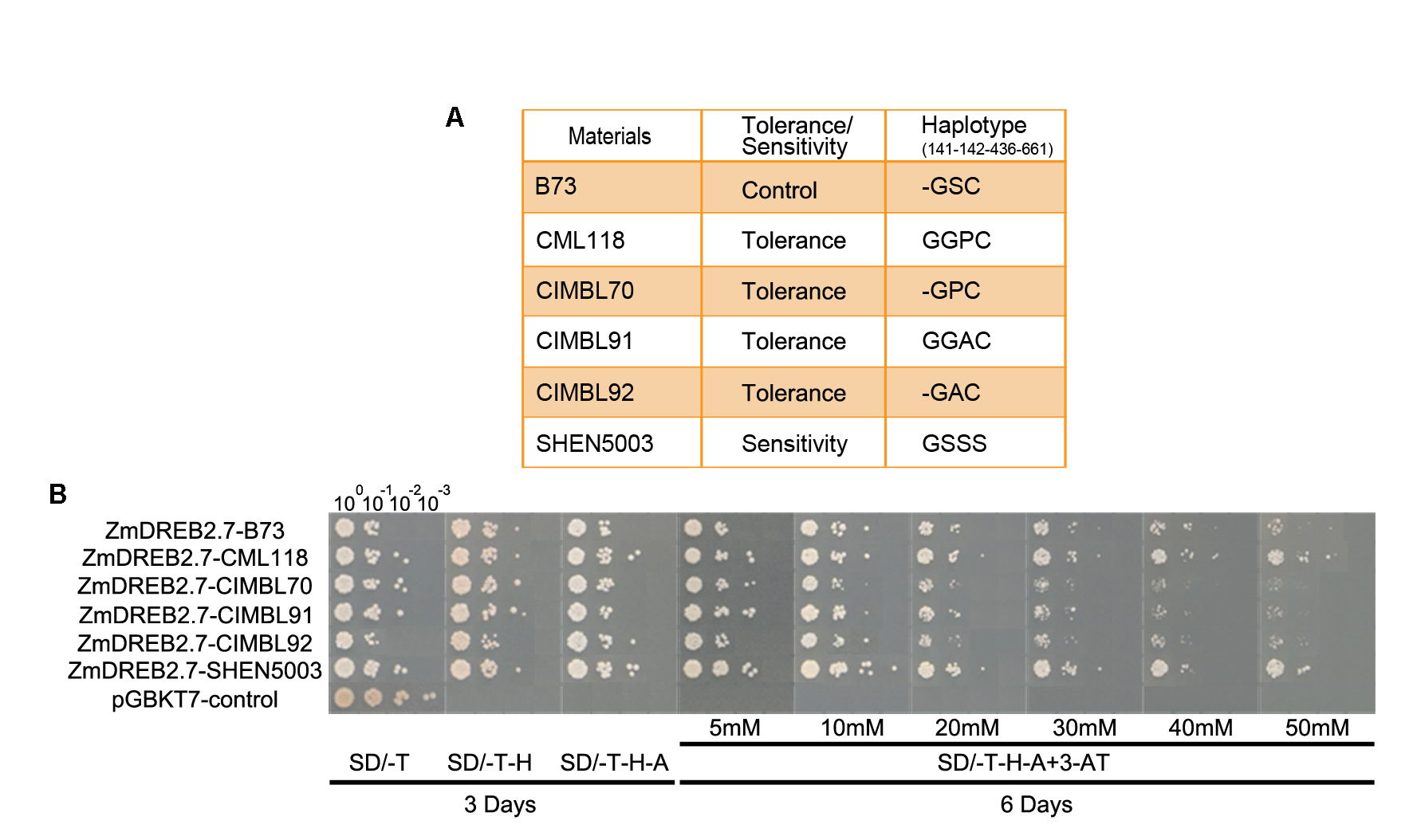

Supplement: Figure S3 — The transactivation activity of ZmDREB2.7 proteins encoded by different haplotypes identified in drought tolerant and sensitive maize varieties. (A) The name of different maize inbred lines and their haplotypes at the four nonsynonymous significant sites in the coding region. (B) Yeast strain AH109 transformed with a vector carrying the ZmDREB2.7 gene which was cloned from CIMBL70, 91, 92, CML118, Shen5003 and B73 inbred lines. Cultures of transformed yeast cells were diluted and placed on agar culture plates containing a -tryptophan (-T) synthetic dropout (SD) medium (SD/-T), -tryptophan-histidine (SD/-T-H) medium, or -tryptophan-histidine-adenine (SD/-T-H-A) medium. The (SD/-T-H-A) medium was amended with different concentrations of 3-aminotriazole (3-AT). (TIF) [file pgen.1003790.s003.tif]

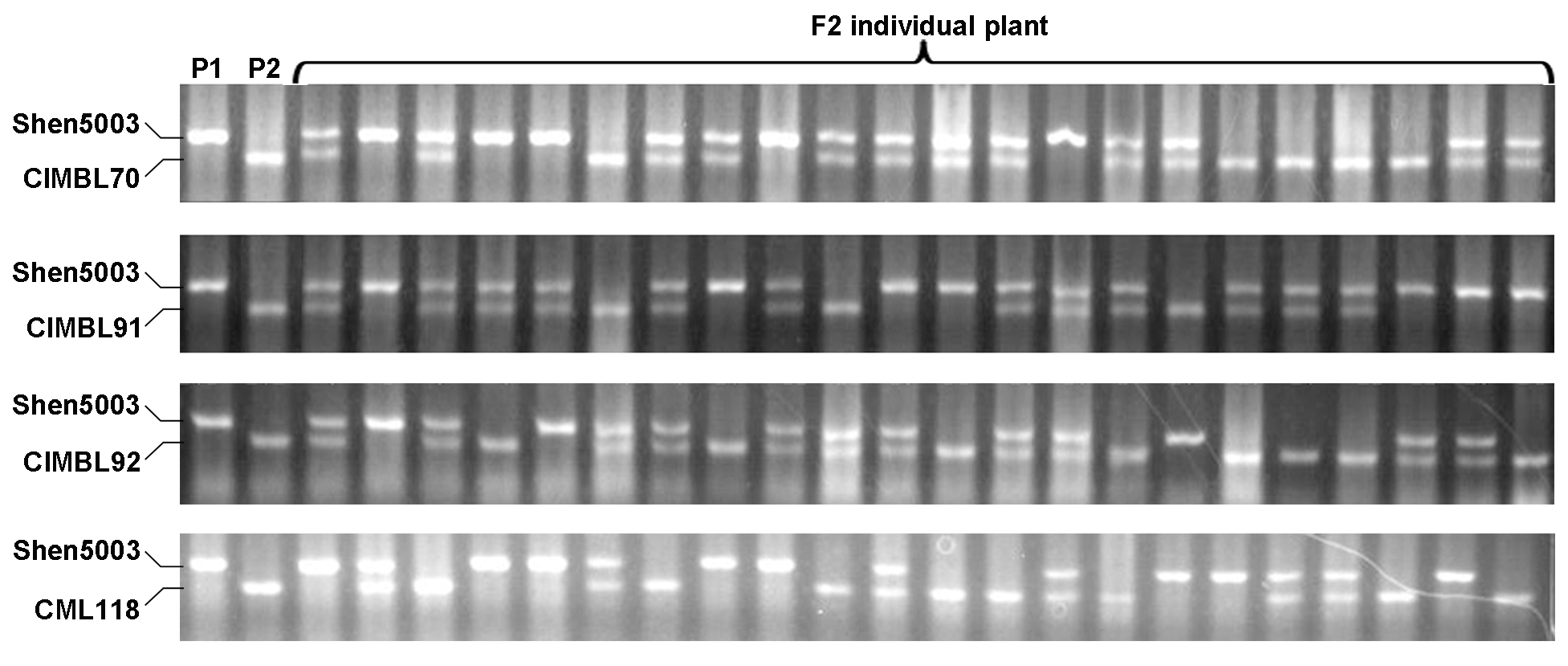

Supplement: Figure S4 — Genotyping of F2 individuals from the four segregating populations. Examples of PCR amplifications of DNA from F2 individuals and their parents of the four F2 populations. PCR amplification utilized primers surrounding the 20-bp InDel polymorphism upstream from the start codon of ZmDREB2.7. The size of the DNA band from Shen5003 was 66-bp and that from CIMBL70, 91, 92 and CML118 was 46-bp. Gel electrophoresis utilizing 3% agar was used to analyze fragments obtained by PCR amplification. (TIF) [file pgen.1003790.s004.tif]

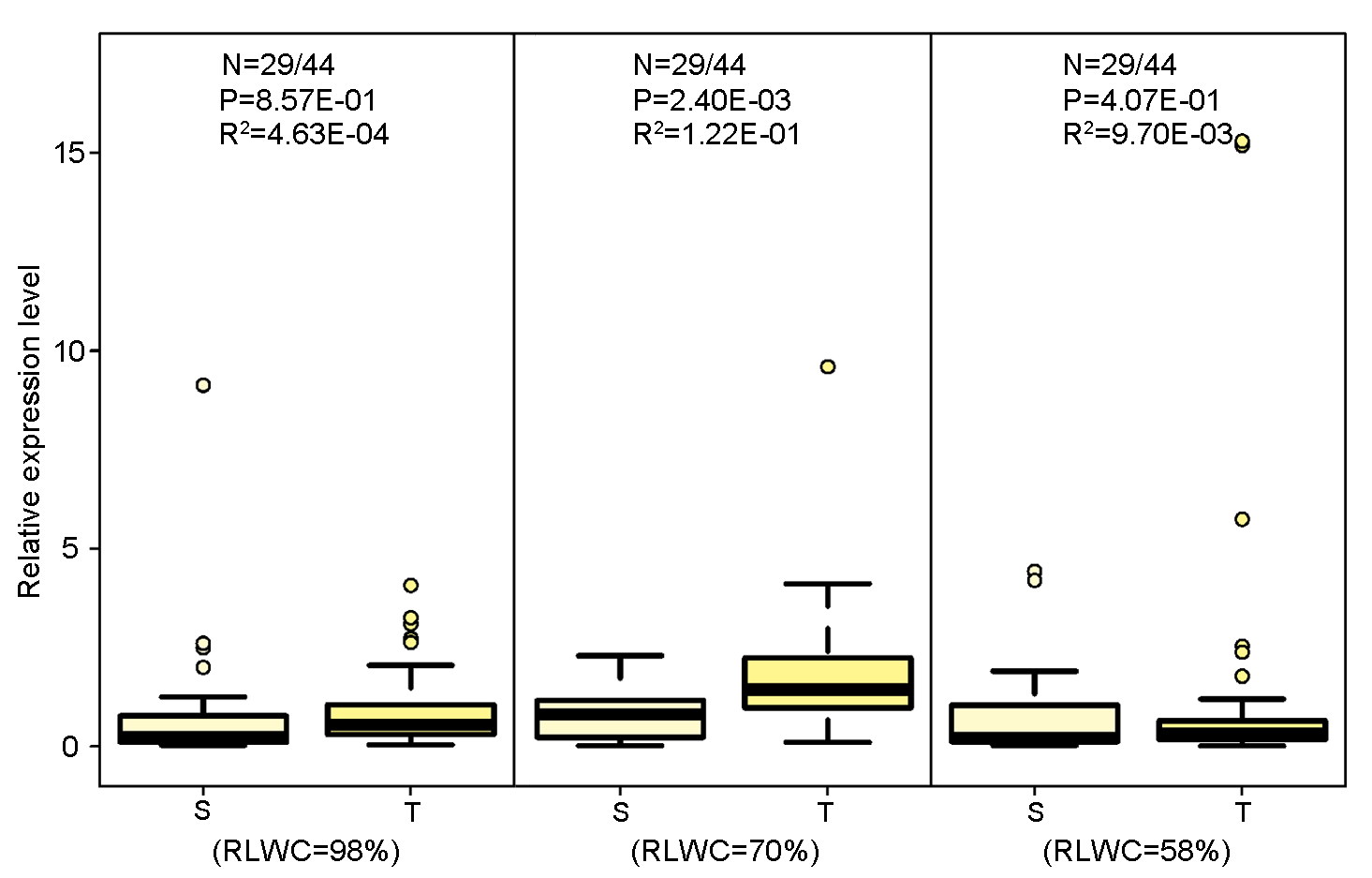

Supplement: Figure S5 — Comparison of ZmDREB2.7 gene expression level between maize inbred lines carrying a ZmDREB2.7 drought tolerant or sensitive allele. The allelic grouping was based on five significant polymorphisms in the 5′-UTR. “T” indicates the tolerant allele, while “S” indicates the sensitive allele. Drought stress was applied to the maize seedlings after the RLWC was decreased from 98% (unstressed) to 70% (moderate drought) or 58% (severe drought). A one-way ANOVA using the lm function in R program (http://www.R-project.org) was applied to analyze the statistical differences of relative gene expression levels in maize seedlings. (TIF) [file pgen.1003790.s005.tif]

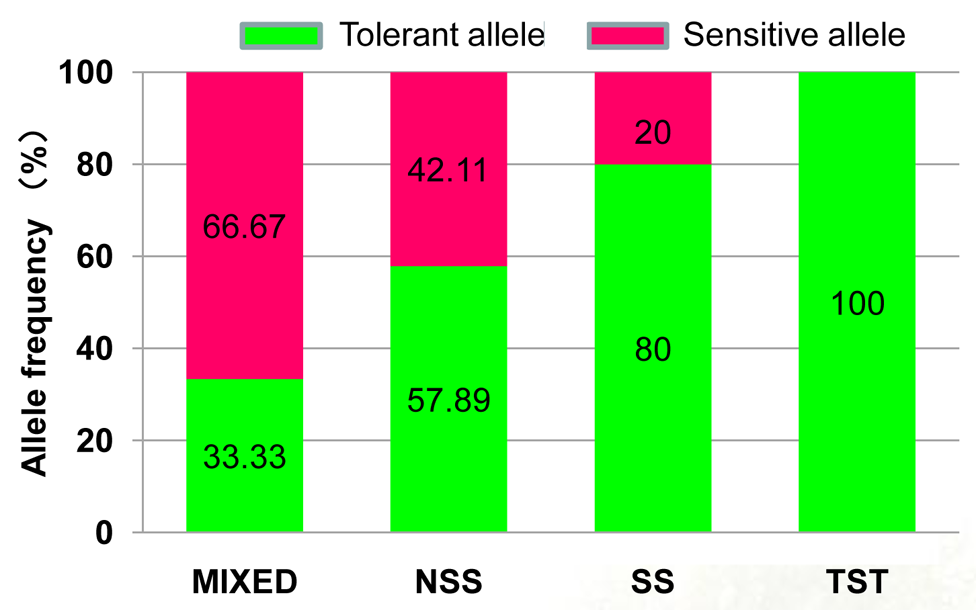

Supplement: Figure S6 — Frequency of the favorable allele of ZmDREB2.7 among different subpopulations. The sequences corresponding to the 5′-UTR of ZmDREB2.7 from 105 randomly selected inbred lines were analyzed. Division of the population into subpopulations (MIXED, NSS, SS, and TST) was according to Yang et al., 2011 [42] where TST = tropical or subtropical varieties; NSS = temperate varieties; SS = B73 derivatives and MIXE = varieties with no clear identity. (TIF) [file pgen.1003790.s006.tif]
